# A new kind of Bernstein-Schurer-Stancu-Kantorovich-type operators based on *q*-integers

**DOI:** 10.1186/s13660-017-1298-y

**Published:** 2017-02-27

**Authors:** Ruchi Chauhan, Nurhayat Ispir, PN Agrawal

**Affiliations:** 1 Department of Mathematics, Indian Institute of Technology Roorkee, Roorkee, 247667 India; 2 Department of Mathematics, Faculty of Sciences, Gazi University, Ankara, 06500 Turkey

**Keywords:** 41A25, 26A15, 41A28, *q*-Bernstein-Schurer-Kantorovich, rate of convergence, modulus of continuity, GBS operators, mixed modulus of continuity

## Abstract

Agrawal *et al.* (Boll. Unione Mat. Ital. 8:169-180, 2015) introduced a Stancu-type Kantorovich modification of the operators proposed by Ren and Zeng (Bull. Korean Math. Soc. 50(4):1145-1156, 2013) and studied a basic convergence theorem by using the Bohman-Korovokin criterion, the rate of convergence involving the modulus of continuity, and the Lipschitz function. The concern of this paper is to obtain Voronoskaja-type asymptotic result by calculating an estimate of fourth order central moment for these operators and discuss the rate of convergence for the bivariate case by using the complete and partial moduli of continuity and the degree of approximation by means of a Lipschitz-type function and the Peetre *K*-functional. Also, we consider the associated GBS (generalized Boolean sum) operators and estimate the rate of convergence for these operators with the help of a mixed modulus of smoothness. Furthermore, we show the rate of convergence of these operators (univariate case) to certain functions with the help of the illustrations using Maple algorithms and in the bivariate case, the rate of convergence of these operators is compared with the associated GBS operators by illustrative graphics.

## Introduction

Following [3], for any fixed real number $q>0$, satisfying the condition $0< q<1$, the *q*-integer $[k]_{q}$, for $k\in\mathbb{N}$ and *q*-factorial $[k]_{q}!$ are defined as

$$ [k]_{q}=\left \{ \textstyle\begin{array}{l@{\quad}l} \frac{(1-q^{k})}{(1-q)}, & \mbox{if }q\neq 1, \\ k ,& \mbox{if }q=1, \end{array}\displaystyle \right . $$

and

$$ [k]_{q}!=\left \{ \textstyle\begin{array}{l@{\quad}l} [k]_{q}[k-1]_{q}\cdots1, & \mbox{if }k\geq1, \\ 1 ,& \mbox{if }k=0, \end{array}\displaystyle \right . $$

respectively. For any integers *n*, *k* satisfying $0\leq k\leq n$, the *q*-binomial coefficient is given by

$$ {n\choose k}_{q}=\frac{[n]_{q}!}{[n-k]_{q}![k]_{q}!}. $$

The *q*-analogue of $( 1-x )^{n}$ is given by

$$ ( 1-x )_{q}^{n}=\left \{ \textstyle\begin{array}{l@{\quad}l} \prod_{j=0}^{n-1} ( 1-q^{j}x ) , & n=1,2,\ldots, \\ 1, & n=0. \end{array}\displaystyle \right . $$

The *q*-integration in the interval $[0,a]$ is defined by

$$\int_{0}^{a} f(t)\, d_{q}t=a(1-q) \sum _{n=0}^{\infty}f\bigl(aq^{n} \bigr)q^{n},\quad 0< q< 1, $$

provided the series converges.

Let $I=[0,1+p]$ and $p\in\mathbb{N}\cup\{0\}$. For $f\in C(I)$, the space of all continuous functions on *I* endowed with the norm $\| f\|= \sup_{x\in[0,1+p]}|f(x)|$ and $0< q<1$, Ren and Zeng [2] defined the following new version of the *q*-Bernstein-Schurer operator which preserves the linear functions:

1.1 $$ \overline{B}_{n}^{p}\bigl(f(t);q,x\bigr)=\sum _{k=0}^{n+p}\tilde{p}^{*}_{n,k}(q,x)f \biggl(\frac{[k]_{q}}{[n]_{q}} \biggr), $$

where

$$\tilde{p}^{*}_{n,k}(q,x)=\frac{[n]_{q}^{n+p}}{[n+p]_{q}^{n+p}}{n+p\choose k}_{q} x^{k} \biggl(\frac{[n+p]_{q}}{[n]_{q}}-x \biggr)_{q}^{n+p-k}. $$

Later, Acu [4] proposed a *q*-Durrmeyer modification of the operators (1.1) as

1.2 $$ D_{n,p}(f;q,x)=\frac{[n+p+1]_{q}[n]_{q}}{[n+p]_{q}}\sum _{k=0}^{n+p}\tilde {p}^{*}_{n,k}(q,x) \int_{0}^{\frac{[n+p]_{q}}{[n]_{q}}}f(t)\tilde{b}_{n,k}^{p}(q,qt) \, d_{q}t $$

and discussed the rate of convergence in terms of the modulus of continuity, a Lipschitz class function, and a Voronovskaja-type result. Subsequently, for $\alpha, \beta\in\mathbb{R}$ such that $0\leq\alpha \leq\beta$ and $f\in C(I)$, Agrawal *et al.* [1] introduced a Stancu-type Kantorovich modification of the operators (1.1), defined as

1.3 $$ \mathcal{K}_{n,p}^{(\alpha,\beta)}(f;q,x)= \sum _{k=0}^{n+p}\tilde{p}^{*}_{n,k}(q,x) \int_{0}^{1}f \biggl(\frac{[k]_{q}+q^{k}t+\alpha}{[n+1]_{q}+\beta} \biggr)\, d_{q}t, $$

and discussed the basic convergence theorem, the rate of convergence involving modulus of continuity and Lipschitz function. Significant contributions have been made by researchers in this area of approximation theory (*cf.* [5] and the references their in).

The purpose of this paper is to discuss the Voronoskaja asymptotic result by calculating an estimate of the fourth order central moment for the operators (1.3) and construct the bivariate case of these operators. We obtain the rate of approximation of the bivariate operators by using the complete and partial moduli of continuity and the degree of approximation with the aid of a Lipschitz-type space and the Peetre *K*-functional. Lastly, we consider the associated GBS (generalized Boolean sum) operators and study the approximation of Bögel continuous and Bögel differentiable functions by means of the mixed modulus of smoothness.

### Lemma 1

[1]

*For the operators given by* (1.3), *the following equalities hold*:

(i) $\mathcal{K}_{n,p}^{(\alpha,\beta)}(1;q,x)=1$;
(ii) $\mathcal{K}_{n,p}^{(\alpha,\beta)}(t;q,x)=\frac{\alpha }{[n+1]_{q}+\beta}+\frac{2q[n]_{q}x+1}{[2]_{q}([n+1]_{q}+\beta)}$;
(iii) $\mathcal{K}_{n,p}^{(\alpha,\beta)}(t^{2};q,x)=\frac {1}{[2]_{q}[3]_{q}([n+1]_{q}+\beta)^{2}} \{\frac {[n]_{q}^{2}[n+p-1]_{q}}{[n+p]_{q}}([3]_{q}q^{2}+3q^{4})x^{2} +\{(4\alpha+3)q[3]_{q}+q^{2}(1+[2]_{q})\}[n]_{q}x +[4]_{q}\alpha^{2}+2\alpha[3]_{q}+(1+q\alpha^{2})[2]_{q} \}$.

### Lemma 2

[1]

*For* $m\in\mathbb{N}\cup\{0\}$, *the* *mth order central moment of* $\mathcal{K}_{n,p}^{(\alpha,\beta)}(f;q,x)$ *defined as* $\mu _{n,m,q}^{*}(x)=\mathcal{K}_{n,p}^{\alpha,\beta}((t-x)^{m};q,x)$, *we have*

(i) $\mu_{n,1,q}^{*}(x)= \frac{(2-[2]_{q})q[n]_{q} x -(\beta+1)[2]_{q} x +1}{[2]_{q}([n+1]_{q}+\beta)}+\frac{\alpha}{[n+1]_{q}+\beta}$;
(ii) $\mu_{n,2,q}^{*}(x)= \{\frac{[n]_{q}^{2}[n+p-1]_{q} ([3]_{q}q^{2}+3q^{4})}{[n+p]_{q} ([n+1]_{q}+\beta)^{2} [2]_{q}[3]_{q}}-\frac{4 q [n]_{q}}{[2]_{q} [n+1]_{q}+\beta}+1 \}x^{2}+ \{\frac{\{(4\alpha +3)[3]_{q} q+q^{2}(1+[2]_{q})\}[n]_{q}}{([n+1]_{q}+\beta)^{2} [2]_{q}[3]_{q}}-\frac {2\alpha}{[n+1]_{q}+\beta}-\frac{2}{[2]_{q}[n+1]_{q}+\beta} \}x+\frac {[4]_{q}\alpha^{2} +2\alpha[3]_{q}+(1+q\alpha^{2})[2]_{q}}{([n+1]_{q}+\beta)^{2}[2]_{q}[3]_{q}}$.

*In the following we obtain an estimate of the fourth order central moment of the operators defined by* (1.3).

*By the definition of the Jackson integral and the inequality* $(a+b)^{4}\leq 8(a^{4}+b^{4})$, *where* $a>0$, $b>0$, *and Lemma *2.4 *in* [2], *we have*

1.4 $$\begin{aligned} \mathcal{K}_{n,p}^{(\alpha,\beta)}\bigl((t-x)^{4};q_{n},x \bigr) =&[n+1]_{q_{n}}\sum_{k=0}^{n+p} \tilde{p}_{n,k}^{*}(q_{n},x) \int_{0}^{1} \biggl(\frac {[k]_{q_{n}}+q_{n}^{k}t+\alpha}{[n+1]_{q_{n}}+\beta}-x \biggr)^{4} \, d_{q_{n}}t \\ =&\sum_{k=0}^{n+p}\tilde{p}_{n,k}^{*}(q_{n},x) (1-q_{n})\sum_{j=0}^{\infty } \biggl( \frac{[k]_{q_{n}}+q_{n}^{k}q_{n}^{j}+\alpha}{[n+1]_{q_{n}}+\beta}-x \biggr)^{4}\times q_{n}^{j} \\ \leq&8\sum_{k=0}^{n+p} \tilde{p}_{n,k}^{*}(q_{n},x) \biggl(\frac {[k]_{q_{n}}+\alpha}{[n+1]_{q_{n}}+\beta}-x \biggr)^{4} \\ &{}+8(1-q_{n})\sum_{k=0}^{n+p} \tilde{p}_{n,k}^{*}(q_{n},x)\sum_{j=0}^{\infty } \biggl(\frac{q_{n}^{k}}{[n+1]_{q_{n}}+\beta} \biggr)^{4}q_{n}^{5j} \\ \leq&64\sum_{k=0}^{n+p}p_{n,k}^{*}(q_{n},x) \biggl(\frac{[k]_{q_{n}}+\alpha }{[n]_{q_{n}}+\beta} \biggr)^{4} \biggl(\frac{q_{n}^{n}}{[n+1]_{q_{n}}+\beta} \biggr)^{4} \\ &{}+64\sum_{k=0}^{n+p} \tilde{p}_{n,k}^{*}(q_{n},x) \biggl(\frac {[k]_{q_{n}}+\alpha}{[n]_{q_{n}}+\beta}-x \biggr)^{4} \\ &{}+\frac {8}{1+q_{n}+q_{n}^{2}+q_{n}^{3}+q_{n}^{4}}\sum_{k=0}^{n+p}\tilde {p}_{n,k}^{*}(q_{n},x) \biggl(\frac{q_{n}^{k}}{[n+1]_{q_{n}}} \biggr)^{4} \\ =&64\sum_{k=0}^{n+p}p_{n,k}^{*}(q_{n},x) \biggl(\frac{[k]_{q_{n}}+\alpha }{[n]_{q_{n}}+\beta} \biggr)^{4} \biggl(\frac{q_{n}^{n}}{[n+1]_{q_{n}}+\beta} \biggr)^{4} \\ &{}+64\overline{B}_{n}^{p}\biggl( \biggl( \frac{[k]_{q_{n}}+\alpha}{[n]_{q_{n}}+\beta }-x \biggr)^{4};q,x\biggr) \\ &{}+\frac {8}{1+q_{n}+q_{n}^{2}+q_{n}^{3}+q_{n}^{4}}\sum_{k=0}^{n+p} \tilde {p}_{n,k}^{*}(q_{n},x) \biggl(\frac{q_{n}^{k}}{[n+1]_{q_{n}}} \biggr)^{4} \\ \leq&64\frac{1}{[n]_{q_{n}}^{2}}+64M_{2}\frac{1/4}{[n]_{q_{n}}^{2}}+ \frac {8}{[n]_{q_{n}}^{2}}=\frac{64+16M_{2}+8}{[n]_{q_{n}}^{2}}. \end{aligned}$$

In the following, let $(q_{n})_{n}$, $0< q_{n}<1$ be a sequence satisfying $\lim_{n\rightarrow\infty}q_{n}=1$ and $\lim_{n\rightarrow\infty}q_{n}^{n}=a$ ($0\leq a<1$).

## Voronovskaja-type theorem

Let $C^{2}[0,1+p]$ denote the space of twice continuously differentiable functions on $[0,1+p]$.

### Theorem 1

*For any* $f\in C^{2}[0,1+p]$,

$$\lim_{n\rightarrow\infty}[n]_{q_{n}}\bigl(\mathcal{K}_{n,p}^{(\alpha,\beta )}(f;q_{n},x)-f(x) \bigr)= \biggl(\frac{-x(a+1+2\beta)}{2}+\alpha+\frac{1}{2} \biggr)f'(x)-x^{2}f''(x) $$

*uniformly in* $[0,1]$.

### Proof

Using Taylor’s expansion for *f*, we obtain

2.1 $$ f(t)=f(x)+f'(x) (t-x)+\frac{f''(x)(t-x)^{2}}{2}+\xi(t,x) (t-x)^{2}, $$

where the function $\xi(t,x)$ is the Peano form of the remainder, $\xi (t,x)\in C[0,1+p]$, and $\lim_{t\rightarrow x}\xi(t,x)=0$.

By linearity of the operators $\mathcal{K}_{n,p}^{(\alpha,\beta )}(;q_{n},x)$ and using Lemma 2, we get

2.2 $$\begin{aligned} \lim_{n\rightarrow\infty}[n]_{q_{n}}\bigl( \mathcal{K}_{n,p}^{(\alpha,\beta )}(f;q_{n},x)-f(x)\bigr) =& \biggl(\frac{-x(a+1+2\beta)}{2}+\alpha+\frac {1}{2} \biggr)f'(x)-x^{2}f''(x) \\ &{}+\lim_{n\rightarrow\infty}[n]_{q_{n}}\mathcal{K}_{n,p}^{(\alpha,\beta )} \bigl(\xi(t,x) (t-x)^{2};q_{n},x\bigr) \end{aligned}$$

uniformly in $[0,1]$.

For the last term of the right side, using the Cauchy-Schwarz inequality, we are led to

$$ [n]_{q_{n}}\mathcal{K}_{n,p}^{(\alpha,\beta)}\bigl(\xi(t,x) (t-x)^{2};q_{n},x\bigr)\leq [n]_{q_{n}}\sqrt {\mathcal{K}_{n,p}^{(\alpha,\beta)}\bigl(\xi ^{2}(t,x);q_{n},x \bigr)}\sqrt{\mathcal{K}_{n,p}^{(\alpha,\beta)} \bigl((t-x)^{4};q_{n},x\bigr)}. $$

We observe that $\xi^{2}(t,x)\in C[0,1+p]$ and $\xi^{2}(x,x)=0$, hence, by Theorem 1

$$ \lim_{n\rightarrow\infty}\mathcal{K}_{n,p}^{(\alpha,\beta)}\bigl(\xi ^{2}(t,x);q_{n},x\bigr)=\xi^{2}(x,x)=0, \quad \mbox{uniformly with respect to } x\in[0,1]. $$

Further using (1.4), $\lim_{n\rightarrow\infty}[n]_{q_{n}}\sqrt {\mathcal{K}_{n,p}^{(\alpha,\beta)}((t-x)^{4};q_{n},x)}$ is finite.

Hence,

2.3 $$ \lim_{n\rightarrow\infty}[n]_{q_{n}} \mathcal{K}_{n,p}^{(\alpha,\beta)}\bigl(\xi (t,x) (t-x)^{2};q_{n},x \bigr)=0 $$

uniformly in $x\in[0,1]$. Finally, consideration of (2.2) and (2.3) completes the proof. □

In the following examples, we illustrate the rate of convergence of the operators given by (1.3) to certain functions.

### Example 1

**Figure 1:**
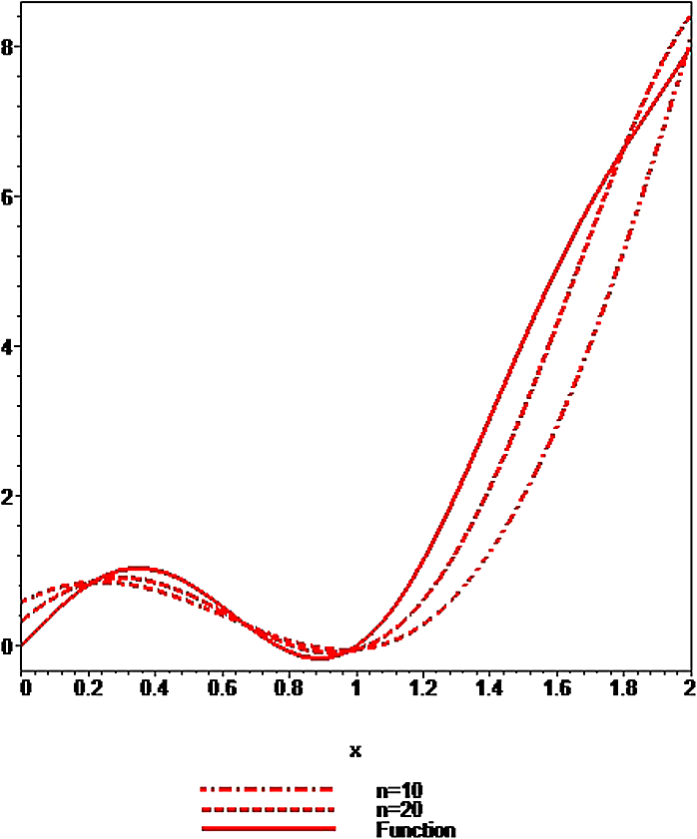
**The convergence of** $\pmb{K_{n,p}^{(\alpha,\beta)}(f;q,x)}$ **to** $\pmb{f(x)}$ **.**

Let $q_{n}=(n-1)/n$. For $\alpha=0.5$, $\beta=0.7$, $p=1$ with $n=10$ and 20, the convergence of $\mathcal{K}_{n,p}^{ ( \alpha,\beta ) }(f;q,x)$ given by (1.3) to $f ( x ) =x^{3}+\sin(3\pi x/2)$ is shown in Figure 1. It is observed that the approximation becomes better on increasing the value of *n*.

### Example 2

**Figure 2:**
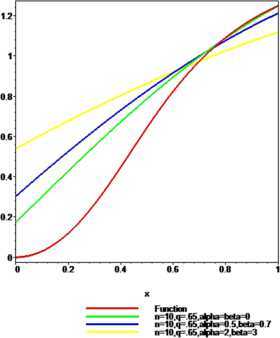
**The convergence of** $\pmb{K_{n,p}^{(\alpha,\beta)}(f;q,x)}$ **to** $\pmb{f(x)}$ **.**

**Figure 3:**
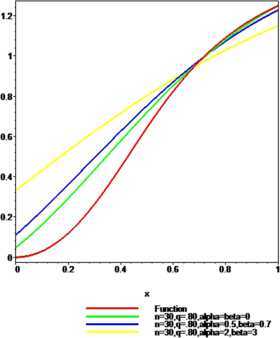
**The convergence of** $\pmb{K_{n,p}^{(\alpha,\beta)}(f;q,x)}$ **to** $\pmb{f(x)}$ **.**

Let $f ( x ) =\arctan(3x^{2})$, $p=0.80$, $n=10$, $q=0.65$ and $n=30$, $q=0.80$. For $\alpha=\beta=0$, $\alpha=0.5$, $\beta=0.7$ and $\alpha=2$, $\beta=3$ the convergence of $\mathcal{K}_{n,p}^{ ( \alpha,\beta ) }(f;q,x)$ to $f(x)$ is shown in Figures 2 and 3 respectively. It is observed that the approximation becomes better when the values of $\alpha,\beta\in [ 0,1 ) $ and the convergence is better in a small interval for larger values of *α*, *β*.

## Construction of the bivariate operators

Let $C(I_{1}\times I_{2})$, where $I_{1}=[0,1+p_{1}]$ and $I_{2}=[0,1+p_{2}]$, denote the space of all real valued continuous functions on $I_{1}\times I_{2}$ endowed with the norm

$$\|f\|_{C(I_{1}\times I_{2})}= \sup_{(x,y)\in I_{1}\times I_{2}}\bigl\vert f(x,y)\bigr\vert . $$

For $f\in C(I_{1}\times I_{2})$, $0< q_{1}$, $q_{2}<1 $ and $J=[0,1]$, the bivariate generalization of the operators given by (1.3) is defined as

3.1 $$\begin{aligned}& \mathcal{K}_{n_{1},n_{2},p_{1},p_{2}}^{(\alpha_{1},\alpha_{2}, \beta_{1},\beta _{2})}\bigl(f(t,s);q_{1},q_{2},x,y \bigr) \\& \quad =\sum_{k_{1}=0}^{n_{1}+p_{1}}\sum _{k_{2}=0}^{n_{2}+p_{2}} \tilde{p}_{n_{1},n_{2},k_{1},k_{2}}^{*}(q_{1},q_{2};x,y) \\& \qquad{} \times \int _{0}^{1} \int_{0}^{1}f\bigl(\Psi_{n_{1},k_{1},q_{1}}^{\alpha_{1},\beta_{1}}(t), \Psi _{n_{2},k_{2},q_{2}}^{\alpha_{2},\beta_{2}}(s)\bigr)\, d_{q_{1}}t\, d_{q_{2}}s, \end{aligned}$$

where

$$\begin{aligned}& \tilde{p}_{n_{1},n_{2},k_{1},k_{2}}^{*}(q_{1},q_{2},x,y) \\& \quad =\frac{[n_{1}]_{q_{1}}^{n_{1}+p_{1}}}{[n_{1}+p_{1}]_{q_{1}}^{n_{1}+p_{1}}}{n_{1}+p_{1} \brack k_{1}}_{q_{1}} x^{k_{1}} \biggl(\frac{[n_{1}+p_{1}]_{q_{1}}}{[n_{1}]_{q_{1}}}-x \biggr)_{q_{1}}^{n_{1}+p_{1}-k_{1}} \\& \qquad {}\times\frac {[n_{2}]_{q_{2}}^{n_{2}+p_{2}}}{[n_{2}+p_{2}]_{q_{2}}^{n_{2}+p_{2}}}{n_{2}+p_{2} \brack k_{2}}_{q_{2}} y^{k_{2}} \biggl(\frac{[n_{2}+p_{2}]_{q_{2}}}{[n_{2}]_{q_{2}}}-y \biggr)_{q_{2}}^{n_{2}+p_{2}-k_{2}}, \quad x,y\in J\quad \mbox{and} \\& \Psi_{n_{1},k_{1},q_{1}}^{\alpha_{1},\beta_{1}}(t)=\frac {[k_{1}]_{q_{1}}+q_{1}^{k_{1}}t+\alpha_{1}}{[n_{1}+1]+\beta_{1}},\qquad \Psi_{n_{2},k_{2},q_{2}}^{\alpha_{2},\beta_{2}}(s)=\frac {[k_{2}]_{q_{2}}+q_{2}^{k_{2}}s+\alpha_{2}}{[n_{2}+1]+\beta_{2}}. \end{aligned}$$

### Lemma 3

*let* $e_{ij}(t,s)=t^{i}s^{j}$, $(t,s)\in(I_{1}\times I_{2})$, $(i,j)\in N^{0}\times N^{0}$ *with* $i+j\leq2$ *be the two dimensional test functions*. *Then the following equalities hold for the operators* (3.1):

(i) $\mathcal{K}_{n_{1},n_{2},p_{1},p_{2}}^{(\alpha_{1},\alpha_{2},\beta_{1},\beta _{2})}(e_{00};q_{1},q_{2},x,y)=1$;
(ii) $\mathcal{K}_{n_{1},n_{2},p_{1},p_{2}}^{(\alpha_{1},\alpha_{2},\beta_{1},\beta _{2})}(e_{10};q_{1},q_{2},x,y)=\frac{\alpha_{1}}{[n_{1}+1]_{q_{1}}+\beta _{1}}+\frac{2q_{1}[n_{1}]_{q_{1}}x+1}{[2]_{q_{1}}([n_{1}+1]_{q_{1}}+\beta_{1})}$;
(iii) $\mathcal{K}_{n_{1},n_{2},p_{1},p_{2}}^{(\alpha_{1},\alpha_{2},\beta_{1},\beta _{2})}(e_{01};q_{1},q_{2},x,y)=\frac{\alpha_{2}}{[n_{2}+1]_{q_{2}}+\beta _{2}}+\frac{2q_{2}[n_{2}]_{q_{2}}y+1}{[2]_{q_{2}}([n_{2}+1]_{q_{2}}+\beta_{2})}$;
(iv) $\mathcal{K}_{n_{1},n_{2},p_{1},p_{2}}^{(\alpha_{1},\alpha_{2},\beta_{1},\beta _{2})}(e_{20};q_{1},q_{2},x,y)=\frac {1}{[2]_{q_{1}}[3]_{q_{1}}([n_{1}+1]_{q_{1}}+\beta_{1})^{2}} \{\frac {[n_{1}]_{q_{1}}^{2}[n_{1}+p_{1}-1]_{q_{1}}}{[n_{1}+p_{1}]_{q_{1}}}([3]_{q_{1}}q_{1}^{2}+3q_{1}^{4})x^{2} +\{(4\alpha_{1}+3)q_{1}[3]_{q_{1}}+q_{1}^{2}(1+[2]_{q_{1}})\}[n_{1}]_{q_{1}}x +[4]_{q_{1}}\alpha_{1}^{2}+2\alpha_{1}[3]_{q_{1}}+(1+q_{1}\alpha_{1}^{2})[2]_{q_{1}} \}$.
(v) $\mathcal{K}_{n_{1},n_{2},p_{1},p_{2}}^{(\alpha_{1},\alpha_{2},\beta_{1},\beta _{2})}(e_{02};q_{1},q_{2},x,y)=\frac {1}{[2]_{q_{2}}[3]_{q_{2}}([n_{2}+1]_{q_{2}}+\beta_{2})^{2}} \{\frac {[n_{2}]_{q_{2}}^{2}[n_{2}+p_{2}-1]_{q_{2}}}{[n_{2}+p_{2}]_{q_{2}}}([3]_{q_{2}}q_{2}^{2}+3q_{2}^{4})y^{2} +\{(4\alpha_{2}+3)q_{2}[3]_{q_{2}}+q_{2}^{2}(1+[2]_{q_{2}})\}[n_{2}]_{q_{2}}y +[4]_{q_{2}}\alpha_{2}^{2}+2\alpha_{2}[3]_{q_{2}}+(1+q_{2}\alpha_{2}^{2})[2]_{q_{2}} \}$.

### Proof

We have $\mathcal{K}_{n_{1},n_{2},p_{1},p_{2}}^{(\alpha_{1},\alpha_{2},\beta_{1},\beta _{2})}(t^{i}s^{j};q_{1},q_{2},x,y)=\mathcal{K}_{n_{1},p_{1}}^{(\alpha_{1},\beta _{1})}(t^{i};q_{1},x) \mathcal{K}_{n_{2},p_{2}}^{(\alpha_{2},\beta _{2})}(s^{j};q_{2},y)$, for $0\leq i,j\leq2$.

By using Lemma 1, the proof of the lemma is straightforward. Hence the details are omitted. □

For $f\in C(I_{1}\times I_{2})$ and $\delta>0 $, the first order complete modulus of continuity for the bivariate case is defined as follows:

$$ \omega(f;\delta_{1},\delta_{2})=\sup \bigl\{ \bigl\vert f(t,s)-f(x,y)\bigr\vert : |t-x|\leq \delta_{1},|s-y|\leq \delta_{2} \bigr\} , $$

where $\delta_{1},\delta_{2}>0$. Further $\omega(f;\delta_{1},\delta_{2})$ satisfies the following properties:

- $\omega(f;\delta_{1},\delta_{2})\rightarrow0 $ if $\delta_{1}\rightarrow 0 $ and $\delta_{2}\rightarrow0$,
- $|f(t,s)-f(x,y)|\leq\omega(f;\delta_{1},\delta_{2}) (1+\frac {|t-x|}{\delta_{1}} ) (1+\frac{|s-y|}{\delta_{2}} )$.

Now, we give an estimate of the rate of convergence of the bivariate operators. In the following, let $0< q_{n_{i}}<1$ be sequences in $(0,1)$ such that $q_{n_{i}}\rightarrow1$ and $q_{n_{i}}^{n_{i}}\rightarrow a_{i}$ ($0\leq a_{i}<1$), as $n_{i}\rightarrow\infty$ for $i=1,2$. Further, let $\delta_{n_{1}}(x)= \mathcal{K}_{n_{1},p_{1}}^{(\alpha_{1},\beta _{1})}((t-x)^{2};q_{n_{1}},x)$ and $\delta_{n_{2}}(y)= \mathcal{K}_{n_{2},p_{2}}^{(\alpha_{2},\beta _{2})}((s-y)^{2};q_{n_{2}},y)$.

### Theorem 2

*For* $f\in C(I_{1}\times I_{2})$ *and all* $(x,y)\in J^{2}$, *we have*

$$ \bigl\vert \mathcal{K}_{n_{1},n_{2},p_{1},p_{2}}^{(\alpha_{1},\alpha_{2}, \beta_{1},\beta _{2})}(f;q_{n_{1}},q_{n_{2}},x,y)-f(x,y) \bigr\vert \leq4\omega\bigl(f;\sqrt{\delta _{n_{1}}(x)},\sqrt{ \delta_{n_{2}}(y)}\bigr). $$

### Proof

Since $\mathcal{K}_{n_{1},n_{2},p_{1},p_{2}}^{(\alpha_{1},\alpha_{2},\beta_{1},\beta _{2})}(f;q_{n_{1}},q_{n_{2}},x,y)$ is a linear positive operator, by the property (b) of bivariate modulus of continuity, Lemma 1, and the Cauchy-Schwarz inequality

$$\begin{aligned}& \bigl\vert \mathcal{K}_{n_{1},n_{2},p_{1},p_{2}}^{(\alpha_{1},\alpha_{2}, \beta_{1},\beta _{2})}\bigl(f(t,s);q_{n_{1}},q_{n_{2}},x,y \bigr)-f(x,y)\bigr\vert \\& \quad \leq \bigl(\mathcal{K}_{n_{1},n_{2},p_{1},p_{2}}^{(\alpha_{1},\alpha_{2}, \beta_{1},\beta _{2})}\bigl\vert f(t,s)-f(x,y)\bigr\vert ;q_{n_{1}},q_{n_{2}},x,y\bigr) \\& \quad \leq \omega\bigl(f;\sqrt{\delta_{n_{1}}(x)},\sqrt{ \delta_{n_{2}}(y)}\bigr) \biggl(\mathcal{K}_{n_{1},p_{1}}^{(\alpha_{1},\beta_{1})}(1;q_{n_{1}},x)+ \frac{1}{\sqrt {\delta_{n_{1}}(x)}}\mathcal{K}_{n_{1},p_{1}}^{(\alpha_{1},\beta _{1})}\bigl(\vert t-x \vert ;q_{n_{1}},x\bigr) \biggr) \\& \qquad {}\times \biggl(\mathcal {K}_{n_{2},p_{2}}^{(\alpha_{2},\beta_{2})}(1;q_{n_{2}},y)+ \frac{1}{\sqrt{\delta _{n_{2}}(y)}} \mathcal{K}_{n_{2},p_{2}}^{(\alpha_{2},\beta _{2})}\bigl(\vert s-y \vert ;q_{n_{2}},y\bigr) \biggr) \\& \quad \leq \omega\bigl(f;\sqrt{\delta_{n_{1}}(x)},\sqrt{ \delta_{n_{2}}(y)}\bigr) \biggl(1+\frac{1}{\sqrt{\delta_{n_{1}}(x)}} \sqrt{\bigl( \mathcal{K}_{n_{1},p_{1}}^{(\alpha_{1},\beta _{1})}(t-x)^{2};q_{n_{1}},x \bigr)} \biggr) \\& \qquad {}\times \biggl(1+\frac{1}{\sqrt{\delta _{n_{2}}(y)}}\sqrt{\mathcal{K}_{n_{2},p_{2}}^{(\alpha_{2},\beta _{2})} \bigl((s-y)^{2};q_{n_{2}},y\bigr)} \biggr), \end{aligned}$$

we get the desired result. □

### Theorem 3

*If* $f(x,y)$ *has continuous partial derivatives* $\frac{\partial f}{\partial x}$ *and* $\frac{\partial f}{\partial y}$, *then the inequality*

$$\begin{aligned}& \bigl\vert \mathcal{K}_{n_{1},n_{2},p_{1},p_{2}}^{(\alpha_{1},\alpha_{2}, \beta_{1},\beta _{2})}(f;q_{n_{1}},q_{n_{2}},x,y)-f(x,y) \bigr\vert \\& \quad \leq M_{1} \lambda_{n_{1}}(x) +\omega \bigl(f'_{x},\delta_{n_{1}}(x)\bigr) \bigl(1+ \sqrt{\delta_{n_{1}}(x)}\bigr) \\& \qquad {}+M_{2} \lambda_{n_{2}}(y)+\omega\bigl(f'_{y}, \delta_{n_{2}}(y)\bigr) \bigl(1+\sqrt{\delta_{n_{2}}(y)}\bigr), \end{aligned}$$

*where* $M_{1}$, $M_{2}$ *are the positive constants such that*

$$ \biggl\vert \frac{\partial f}{\partial x}\biggr\vert \leq M_{1},\qquad \biggl\vert \frac{\partial f}{\partial y}\biggr\vert \leq M_{2} \quad (0\leq x \leq a, 0\leq y\leq b) $$

*and*

$$\begin{aligned}& \lambda_{n_{1}}(x)= \biggl\vert \frac{(2-[2]_{q_{n_{1}}})q_{n_{1}}[n_{1}]_{q_{n_{1}}} -(\beta_{1}+1)[2]_{q_{n_{1}}}}{[2]_{q_{n_{1}}}([n_{1}+1]_{q_{n_{1}}}+\beta _{1})} \biggr\vert x+ \frac{(1+[2]_{q_{n_{1}}}\alpha_{1})}{[n_{1}+1]_{q_{n_{1}}}+\beta _{1}} ; \\& \lambda_{n_{2}}(y)= \biggl\vert \frac {(2-[2]_{q_{n_{2}}})q_{n_{2}}[n_{2}]_{q_{n_{2}}}-(\beta _{2}+1)[2]_{q_{n_{2}}}}{[2]_{q_{n_{2}}}([n_{2}+1]_{q_{n_{2}}}+\beta_{2})} \biggr\vert y + \frac{(1+[2]_{q_{n_{2}}}\alpha_{2})}{[2]_{q_{n_{2}}}([n_{2}+1]_{q_{n_{2}}}+\beta_{2})} . \end{aligned}$$

### Proof

From the mean value theorem we have

3.2 $$\begin{aligned} f(t,s)-f(x,y) =&f(t,y)-f(x,y)+f(t,s)-f(t,y) \\ =&(t-x)\frac{\partial f(\xi_{1},y)}{\partial x}+(s-y)\frac{\partial f(x,\xi_{2})}{\partial y} \\ =&(t-x)\frac{\partial f(x,y)}{\partial x}+(t-x) \biggl(\frac{\partial f(\xi_{1},y)}{\partial x}-\frac{\partial f(x,y)}{\partial x} \biggr)+(s-y)\frac{\partial f(x,y)}{\partial y} \\ &{}+(s-y) \biggl(\frac {\partial f(x,\xi_{2})}{\partial y}-\frac{\partial f(x,y)}{\partial y} \biggr), \end{aligned}$$

where $x <\xi< t$ and $y<\xi_{2}<s$. Since

$$\begin{aligned}& \biggl\vert \frac{\partial f(\xi_{1},y)}{\partial x}-\frac{\partial f(x,y)}{\partial x}\biggr\vert \leq\omega \bigl(f'_{x};\vert t-x\vert \bigr)\leq \biggl(1+ \frac {\vert t-x\vert }{\delta_{n_{1}}} \biggr)\omega\bigl(f'_{x}, \delta_{n_{1}}\bigr)\quad \mbox{and} \\& \biggl\vert \frac{\partial f(x,\xi_{2})}{\partial y}-\frac{\partial f(x,y)}{\partial y}\biggr\vert \leq\omega \bigl(f'_{y};\vert s-y\vert \bigr)\leq \biggl(1+ \frac {\vert s-y\vert }{\delta_{n_{2}}} \biggr)\omega\bigl(f'_{y}, \delta_{n_{2}}\bigr) \end{aligned}$$

for some $\delta_{n_{1}},\delta_{n_{2}}>0$, on applying the operator $\mathcal{K}_{n_{1},n_{2},p_{1},p_{2}}^{(\alpha_{1},\alpha_{2},\beta_{1},\beta _{2})}(\cdot;q_{n_{1}},q_{n_{2}},x,y)$ on both sides of (3.2), we have

$$\begin{aligned}& \bigl\vert \mathcal{K}_{n_{1},n_{2},p_{1},p_{2}}^{(\alpha_{1},\alpha_{2}, \beta_{1},\beta _{2})}(f;q_{n_{1}},q_{n_{2}},x,y)-f(x,y) \bigr\vert \\& \quad \leq M_{1}\bigl\vert \mathcal{K}_{n_{1},p_{1}}^{(\alpha_{1},\beta _{1})}(e_{10}-x;q_{n_{1}},x) \bigr\vert \\& \qquad {}+\sum_{k_{1}=0}^{n_{1}+p_{1}}\sum _{k_{2}=0}^{n_{2}+p_{2}} \tilde{p}_{n_{1},n_{2},k_{1},k_{2}}^{*}(q_{n_{1}},q_{n_{2}};x,y) \\& \qquad {}\times \int_{0}^{1} \int _{0}^{1}\bigl\vert \Psi_{n_{1},k_{1},q_{n_{1}}}^{\alpha_{1},\beta_{1}}(t)-x \bigr\vert \omega \bigl(f'_{x},\delta_{n_{1}} \bigr) \biggl(\frac{\vert \Psi_{n_{1},k_{1},q_{n_{1}}}^{\alpha_{1},\beta_{1}}(t)-x\vert }{\delta _{n_{1}}}+1 \biggr)\, d_{q_{n_{1}}}t\, d_{q_{n_{2}}}s \\& \qquad {}+M_{2}\bigl\vert \mathcal{K}_{n_{2},p_{2},q_{n_{2}}}^{(\alpha_{2},\beta_{2})}(e_{01}-y;y) \bigr\vert \\& \qquad {}+\sum_{k_{1}=0}^{n_{1}+p_{1}}\sum _{k_{2}=0}^{n_{2}+p_{2}} \tilde{p}_{n_{1},n_{2},k_{1},k_{2}}^{*}(q_{n_{1}},q_{n_{2}};x,y) \\& \qquad {}\times \int_{0}^{1} \int _{0}^{1}\bigl\vert \Psi_{n_{2},k_{2},q_{n_{2}}}^{\alpha_{2},\beta_{2}}(s)-y \bigr\vert \omega \bigl(f'_{y},\delta_{n_{2}} \bigr) \biggl(\frac{\vert \Psi_{n_{2},k_{2},q_{n_{2}}}^{\alpha_{2},\beta_{2}}(s)-y\vert }{\delta _{n_{2}}}+1 \biggr)\, d_{q_{n_{1}}}t\, d_{q_{n_{2}}}s. \end{aligned}$$

Now applying the Cauchy-Schwarz inequality

$$\begin{aligned}& \bigl\vert \mathcal{K}_{n_{1},n_{2},p_{1},p_{2}}^{(\alpha_{1},\alpha_{2}, \beta_{1},\beta _{2})}(f;q_{n_{1}},q_{n_{2}},x,y)-f(x,y) \bigr\vert \\& \quad \leq M_{1}\bigl\vert \mathcal{K}_{n_{1},p_{1}}^{(\alpha_{1},\beta _{1})}(e_{10};q_{n_{1}},x) \bigr\vert \\& \qquad {}+ \omega\bigl(f'_{x},\delta_{n_{1}} \bigr) \Biggl\{ \sum_{k_{1}=0}^{n_{1}+p_{1}}\sum _{k_{2}=0}^{n_{2}+p_{2}} \tilde{p}_{n_{1},n_{2},k_{1},k_{2}}^{*}(q_{n_{1}},q_{n_{2}};x,y) \\& \qquad {}\times \int_{0}^{1} \int _{0}^{1}\bigl(\Psi_{n_{1},k_{1},q_{n_{1}}}^{\alpha_{1},\beta_{1}}(t)-x \bigr)^{2}\,d_{q_{n_{1}}}t \,d_{q_{n_{2}}}s \Biggr\} ^{\frac{1}{2}} \\& \qquad {}+\frac{\omega(f'_{x},\delta_{n_{1}})}{ \delta_{n_{1}}}\sum_{k_{1}=0}^{n_{1}+p_{1}} \sum_{k_{2}=0}^{n_{2}+p_{2}} \tilde{p}_{n_{1},n_{2},k_{1},k_{2}}^{*}(q_{n_{1}},q_{n_{2}};x,y) \\& \qquad {}\times \int_{0}^{1} \int _{0}^{1}\bigl(\Psi_{n_{1},k_{1},q_{n_{1}}}^{\alpha_{1},\beta_{1}}(t)-x \bigr)^{2}\,d_{q_{n_{1}}}t \,d_{q_{n_{2}}}s \\& \qquad {}+M_{2}\bigl\vert \mathcal{K}_{n_{2},p_{2}}^{(\alpha_{2},\beta_{2})}(e_{01};q_{n_{2}},y) \bigr\vert \\& \qquad {}+\omega\bigl(f'_{y},\delta_{n_{2}} \bigr) \Biggl\{ \sum_{k_{1}=0}^{n_{1}+p_{1}}\sum _{k_{2}=0}^{n_{2}+p_{2}} \tilde{p}_{n_{1},n_{2},k_{1},k_{2}}^{*}(q_{n_{1}},q_{n_{2}};x,y) \\& \qquad {}\times \int_{0}^{1} \int _{0}^{1}\bigl(\Psi_{n_{2},k_{2},q_{n_{2}}}^{\alpha_{2},\beta_{2}}(s)-y \bigr)^{2}\,d_{q_{n_{1}}}t \,d_{q_{n_{2}}}s \Biggr\} ^{\frac{1}{2}} \\& \qquad {}+\frac{\omega(f'_{y},\delta_{n_{2}})}{ \delta_{n_{2}}}\sum_{k_{1}=0}^{n_{1}+p_{1}} \sum_{k_{2}=0}^{n_{2}+p_{2}} \tilde{p}_{n_{1},n_{2},k_{1},k_{2}}^{*}(q_{n_{1}},q_{n_{2}};x,y) \\& \qquad {}\times \int_{0}^{1} \int _{0}^{1}\bigl(\Psi_{n_{2},k_{2},q_{n_{2}}}^{\alpha_{2},\beta_{2}}(s)-y \bigr)^{2}\,d_{q_{n_{1}}}t \,d_{q_{n_{2}}}s \\& \quad = M_{1} \lambda_{n_{1}}(x)+\omega\bigl(f'_{x}, \delta_{n_{1}}\bigr) (1+\sqrt{\delta _{n_{1}}})+M_{2} \lambda_{n_{2}}(y)+\omega\bigl(f'_{y}, \delta_{n_{2}}\bigr) (1+\sqrt{\delta _{n_{2}}}), \end{aligned}$$

on choosing $\delta_{n_{1}}=\delta_{n_{1}}(x)$ and $\delta_{n_{2}}=\delta_{n_{2}}(y)$, we obtain the required result. □

### Degree of approximation

In our next result, we study the degree of approximation for the bivariate operators by means of the Lipschitz class.

For $0<\xi_{1}\leq1 $ and $0<\xi_{2}\leq1 $, we define the Lipschitz class $\operatorname{Lip}_{M}(\xi_{1},\xi_{2})$ for the bivariate case as follows:

$$ \bigl\vert f(t,s)-f(x,y)\bigr\vert \leq M|t-x|^{\xi_{1}}|s-y|^{\xi_{2}}, $$

where $(t,s), (x,y)\in(I_{1}\times I_{2})$ are arbitrary.

#### Theorem 4

*Let* $f\in \operatorname{Lip}_{M}(\xi_{1},\xi_{2}) $. *Then*, *for all* $(x,y)\in J^{2}$, *we have*

$$ \bigl\vert \mathcal{K}_{n_{1},n_{2},p_{1},p_{2}}^{(\alpha_{1},\alpha_{2}, \beta_{1},\beta _{2})}(f;q_{n_{1}},q_{n_{2}},x,y)-f(x,y) \bigr\vert \leq M \bigl(\delta_{n_{1}}(x)\bigr)^{\frac{\xi _{1}}{2}}\bigl( \delta_{n_{2}}(y)\bigr)^{\frac{\xi_{2}}{2}}. $$

#### Proof

By our hypothesis, we can write

$$\begin{aligned}& \bigl\vert \mathcal{K}_{n_{1},n_{2},p_{1},p_{2}}^{(\alpha_{1},\alpha_{2}, \beta_{1},\beta _{2})}(f;q_{n_{1}},q_{n_{2}},x,y)-f(x,y) \bigr\vert \\& \quad \leq \mathcal {K}_{n_{1},n_{2},p_{1},p_{2}}^{(\alpha_{1},\alpha_{2}, \beta_{1},\beta _{2})}\bigl(\bigl\vert f(t,s)-f(x,y)\bigr\vert ;q_{n_{1}},q_{n_{2}},x,y\bigr) \\& \quad \leq M \mathcal{K}_{n_{1},n_{2},p_{1},p_{2}}^{(\alpha_{1},\alpha_{2}, \beta _{1},\beta_{2})}\bigl(\vert t-x\vert ^{\xi_{1}}\vert s-y\vert ^{\xi_{2}};q_{n_{1}},q_{n_{2}},x,y \bigr) \\& \quad = M \bigl(\mathcal{K}_{n_{1},p_{1}}^{(\alpha_{1},\beta_{1})}\vert t-x\vert ^{\xi _{1}};q_{n_{1}},x\bigr)\mathcal{K}_{n_{2},p_{2}}^{(\alpha_{2},\beta_{2})} \bigl(\vert s-y\vert ^{\xi _{2}};q_{n_{2}},y\bigr). \end{aligned}$$

Now, applying the Hölder’s inequality with $u_{1}=\frac{2}{\xi_{1}}$, $v_{1}=\frac{2}{2-\xi_{1}}$ and $u_{2}=\frac{2}{\xi_{2}}$ and $v_{2}=\frac{2}{2-\xi_{2}}$, respectively, we have

$$\begin{aligned}& \bigl\vert \mathcal{K}_{n_{1},n_{2},p_{1},p_{2}}^{(\alpha_{1},\alpha_{2}, \beta_{1},\beta _{2})}(f;q_{n_{1}},q_{n_{2}},x,y)-f(x) \bigr\vert \\& \quad \leq M {\bigl(\mathcal {K}_{n_{1},p_{1}}^{(\alpha_{1},\beta_{1})}(t-x)^{2};q_{n_{1}},x \bigr)}^{\frac{\xi _{1}}{2}}{\mathcal{K}_{n_{1},p_{1}}^{(\alpha_{1},\beta_{1})}(1;q_{n_{1}},x)}^{\frac {2-\xi_{1}}{2}} \\& \qquad {}\times{\mathcal{K}_{n_{2},p_{2}}^{(\alpha_{2},\beta _{2})}\bigl((s-y)^{2};q_{n_{2}},y \bigr)}^{\frac{\xi_{2}}{2}}{\mathcal{K}_{n_{2},p_{2}}^{\alpha _{2},\beta_{2}}(1;q_{n_{2}},y)}^{\frac{2-\xi_{2}}{2}} \\& \quad = M \bigl(\delta_{n_{1}}(x)\bigr)^{\frac{\xi_{1}}{2}}\bigl( \delta_{n_{2}}(y)\bigr)^{\frac{\xi_{2}}{2}}. \end{aligned}$$

Hence, the proof is completed. □

Let $C^{1}(I_{1}\times I_{2})$ denote the space of all continuous functions on $I_{1}\times I_{2}$ such that their first partial derivatives are continuous on $I_{1}\times I_{2}$.

#### Theorem 5

*For* $f\in C^{1}(I_{1}\times I_{2})$ *and* $(x,y)\in J^{2} $ *we have*

$$ \bigl\vert \mathcal{K}_{n_{1},n_{2},p_{1},p_{2}}^{(\alpha_{1},\alpha_{2}, \beta_{1},\beta _{2})}(f;q_{n_{1}},q_{n_{2}},x,y)-f(x,y) \bigr\vert \leq\bigl\Vert f'_{x}\bigr\Vert _{C(I_{1}\times I_{2})}\sqrt{\delta_{n_{1}}(x)}+\bigl\Vert f'_{y}\bigr\Vert _{C(I_{1}\times I_{2})}\sqrt{ \delta_{n_{2}}(y)}. $$

#### Proof

Let $(x,y)\in J^{2} $ be a fixed point. Then by our hypothesis

$$ f(t,s)-f(x,y)= \int_{x}^{t}f'_{u}(u,s)\, d_{q} u+ \int_{y}^{s}f'_{v}(x,v) \, d_{q} v. $$

Now, operating by $\mathcal{K}_{n_{1},n_{2},p_{1},p_{2}}^{(\alpha_{1},\alpha_{2}, \beta_{1},\beta_{2})}(\cdot;q_{n_{1}},q_{n_{2}},x,y) $ on both sides of the above equation, we are led to

$$\begin{aligned}& \bigl\vert \mathcal{K}_{n_{1},n_{2},p_{1},p_{2}}^{(\alpha_{1},\alpha_{2}, \beta_{1},\beta _{2})}(f;q_{n_{1}},q_{n_{2}},x,y)-f(x,y) \bigr\vert \\& \quad \leq \mathcal {K}_{n_{1},n_{2},p_{1},p_{2}}^{(\alpha_{1},\alpha_{2}, \beta_{1},\beta_{2})} \biggl(\biggl\vert \int _{t}^{x}\bigl\vert f'_{u}(u,s) \bigr\vert \, d_{q} u\biggr\vert ;q_{n_{1}},q_{n_{2}},x,y \biggr) \\& \qquad {}+\mathcal {K}_{n_{1},n_{2},p_{1},p_{2}}^{(\alpha_{1},\alpha_{2},\beta_{1},\beta_{2})} \biggl(\biggl\vert \int _{y}^{s}\bigl\vert f'_{v}(x,v) \bigr\vert \, d_{q} v\biggr\vert ;q_{n_{1}},q_{n_{2}},x,y \biggr). \end{aligned}$$

Since $|\int_{t}^{x}|f'_{u}(u,s)|\, d_{q} u|\leq\| f'_{x}\| _{C(I_{1}\times I_{2})}|t-x|$ and $|\int_{y}^{s}|f'_{v}(x,v)|\, d_{q} v|\leq \| f'_{y}\|_{C(I_{1}\times I_{2})}|s-y|$, we have

$$\begin{aligned}& \bigl\vert \mathcal{K}_{n_{1},n_{2},p_{1},p_{2}}^{(\alpha_{1},\alpha_{2}, \beta_{1},\beta _{2})}(f;q_{n_{1}},q_{n_{2}},x,y)-f(x,y) \bigr\vert \\& \quad \leq\bigl\Vert f'_{x}\bigr\Vert _{C(I_{1}\times I_{2})}\mathcal{K}_{n_{1},p_{1}}^{(\alpha_{1}, \beta_{1})}\bigl(\vert t-x \vert ;q_{n_{1}},x\bigr)+\bigl\Vert f'_{y} \bigr\Vert _{C(I_{1}\times I_{2})}\mathcal{K}_{n_{2},p_{2}}^{(\alpha_{2},\beta _{2})}\bigl( \vert s-y\vert ;q_{n_{2}},y\bigr). \end{aligned}$$

Applying the Cauchy-Schwarz inequality and Lemma 1, we have

$$\begin{aligned}& \bigl\vert \mathcal{K}_{n_{1},n_{2},p_{1},p_{2}}^{(\alpha_{1},\alpha_{2}, \beta_{1},\beta _{2})}(f;q_{n_{1}},q_{n_{2}},x,y)-f(x,y) \bigr\vert \\& \quad \leq \bigl\Vert f'_{x}\bigr\Vert _{C(I_{1}\times I_{2})}\sqrt{\mathcal{K}_{n_{1},p_{1}}^{(\alpha_{1}, \beta_{1})} \bigl((t-x)^{2};q_{n_{1}},x\bigr)} \sqrt{ \mathcal{K}_{n_{1},p_{1}}^{(\alpha_{1},\beta_{1})}(1;q_{n_{1}},x)} \\& \qquad {}+\bigl\Vert f'_{y}\bigr\Vert _{C(I_{1}\times I_{2})} \sqrt{\mathcal{K}_{n_{2},p_{2}}^{(\alpha_{2},\beta _{2})}\bigl((s-y)^{2};q_{n_{2}},y \bigr)}\sqrt{\mathcal{K}_{n_{2},p_{2}}^{(\alpha_{2},\beta _{2})}(1;q_{n_{2}},y)} \\& \quad = \bigl\Vert f'_{x}\bigr\Vert _{C(I_{1}\times I_{2})} \sqrt{\delta _{n_{1}}(x)}+\bigl\Vert f'_{y} \bigr\Vert _{C(I_{1}\times I_{2})}\sqrt{\delta_{n_{2}}(y)}. \end{aligned}$$

This completes the proof of the theorem. □

For $f\in C(I_{1}\times I_{2})$ and $\delta>0$, the partial moduli of continuity with respect to *x* and *y* are given by

$$ \bar{\omega}_{1}(f;\delta)=\sup \bigl\{ \bigl\vert f(x_{1},y)-f(x_{2},y)\bigr\vert :y\in I_{2} \mbox{ and } \vert x_{1}-x_{2}\vert \leq\delta \bigr\} $$

and

$$ \bar{\omega}_{2}(f;\delta)=\sup \bigl\{ \bigl\vert f(x,y_{1})-f(x,y_{2})\bigr\vert :x\in I_{1} \mbox{ and } \vert y_{1}-y_{2}\vert \leq\delta \bigr\} . $$

Clearly, both moduli of continuity satisfy the properties of the usual modulus of continuity.

#### Theorem 6

*If* $f\in C(I_{1}\times I_{2})$ *and* $(x,y)\in J^{2}$, *then we have*

$$ |\mathcal{K}_{n_{1},n_{2},p_{1},p_{2}}^{(\alpha_{1},\alpha_{2}, \beta_{1},\beta _{2})}(f;q_{n_{1}},q_{n_{2}},x,y)-f(x,y)| \leq2\bigl\{ \bigl(\bar{\omega}_{1}\bigl(f;\sqrt{\delta _{n_{1}}(x)}\bigr)\bigr)+\bigl(\bar{\omega}_{2}\bigl(f;\sqrt{ \delta_{n_{2}}(y)}\bigr)\bigr)\bigr\} . $$

#### Proof

Using the definition of partial moduli of continuity, Lemma 1, and the Cauchy-Schwarz inequality, we have

$$\begin{aligned}& \bigl\vert \mathcal{K}_{n_{1},n_{2},p_{1},p_{2}}^{(\alpha_{1},\alpha_{2}, \beta_{1},\beta _{2})}(f;q_{n_{1}},q_{n_{2}},x,y)-f(x,y) \bigr\vert \\& \quad \leq \mathcal {K}_{n_{1},n_{2},p_{1},p_{2}}^{(\alpha_{1},\alpha_{2},\beta_{1},\beta _{2})}\bigl(\bigl\vert f(t,s)-f(x,y)\bigr\vert ;q_{n_{1}},q_{n_{2}},x,y\bigr) \\& \quad \leq \mathcal{K}_{n_{1},n_{2},p_{1},p_{2}}^{(\alpha_{1},\alpha_{2},\beta_{1},\beta _{2})}\bigl(\bigl\vert f(t,s)-f(t,y)\bigr\vert ;q_{n_{1}},q_{n_{2}},x,y\bigr) \\& \qquad {}+\mathcal {K}_{n_{1},n_{2},p_{1},p_{2}}^{(\alpha_{1},\alpha_{2}, \beta_{1},\beta _{2})}\bigl(\bigl\vert f(t,y)-f(x,y)\bigr\vert ;q_{n_{1}},q_{n_{2}},x,y\bigr) \\& \quad \leq \bar{\omega}_{1}\bigl(f;\sqrt{\delta_{n_{1}}(x)} \bigr) \biggl(\mathcal {K}_{n_{1},p_{1}}^{(\alpha_{1},\beta_{1})}(1;q_{n_{1}},x) + \frac{1}{\sqrt{\delta_{n_{1}}(x)}}\mathcal{K}_{n_{1},p_{1}}^{(\alpha_{1},\beta _{1})}\bigl(\vert t-x \vert ;q_{n_{1}},x\bigr) \biggr) \\& \qquad {}+\bar{\omega}_{2}\bigl(f;\sqrt{\delta _{n_{2}}(y)} \bigr) \biggl(\mathcal{K}_{n_{2},p_{2}}^{(\alpha_{2},\beta_{2})}(1;q_{n_{2}},y)+ \frac{1}{\sqrt{\delta_{n_{2}}(y)}}\mathcal{K}_{n_{2},p_{2}}^{(\alpha_{2},\beta _{2})}\bigl(\vert s-y \vert ;q_{n_{2}},y\bigr) \biggr) \\& \quad \leq \bar{\omega}_{1}\bigl(f;\sqrt{\delta_{n_{1}}(x)} \bigr) \biggl(1+\frac{1}{\sqrt {\delta_{n_{1}}(x)}}\sqrt{\mathcal{K}_{n_{1},p_{1}}^{(\alpha_{1},\beta _{1})} \bigl((t-x)^{2};q_{n_{1}},x\bigr)} \biggr) \\& \qquad {}+ \bar{\omega}_{2}\bigl(f;\sqrt{\delta_{n_{2}}(y)} \bigr) \biggl(1+\frac{1}{\sqrt{\delta _{n_{2}}(y)}}\sqrt{\mathcal{K}_{n_{2},p_{2}}^{(\alpha_{2},\beta _{2})} \bigl((s-y)^{2};q_{n_{2}},y\bigr)} \biggr), \end{aligned}$$

from which the required result is straightforward. □

Let $C^{2}(I_{1}\times I_{2})$ be the space of all functions $f\in C(I_{1}\times I_{2})$ such that second partial derivatives of *f* belong to $C(I_{1}\times I_{2})$. The norm on the space $C^{2}(I_{1}\times I_{2}) $ is defined as

$$ \Vert f\Vert _{C^{2}(I_{1}\times I_{2})}=\Vert f\Vert +\sum _{i=1}^{2}\biggl(\biggl\Vert \frac {\partial^{i}f}{\partial x^{i}} \biggr\Vert +\biggl\Vert \frac{\partial^{i}f}{\partial y^{i}}\biggr\Vert \biggr). $$

The Peetre *K*-functional of the function $f\in C(I_{1}\times I_{2})$ is defined as

$$ \mathcal{K}(f;\delta)=\inf_{g\in{C^{2}(I_{1}\times I_{2})}}\bigl\{ \Vert f-g\Vert _{C(I_{1}\times I_{2})}+\delta \Vert g\Vert _{C^{2}(I_{1}\times I_{2})}\bigr\} ,\quad \delta>0. $$

Also by [6], it follows that

3.3 $$ \mathcal{K}(f;\delta)\leq M \bigl\{ \tilde{\omega}_{2}(f; \sqrt{\delta })+\min(1,\delta)\|f\|_{C(I_{1}\times I_{2})} \bigr\} $$

holds for all $\delta>0$.

The constant *M* in the above inequality is independent of *δ* and *f* and $\tilde{\omega}_{2}(f;\sqrt{\delta})$ is the second order modulus of continuity.

#### Theorem 7

*For the function* $f\in C(I_{1}\times I_{2})$, *we have the following inequality*:

$$\begin{aligned}& \bigl\vert \mathcal{K}_{n_{1},n_{2},p_{1},p_{2}}^{(\alpha_{1},\alpha_{2}, \beta _{1},\beta_{2})}(f;q_{n_{1}},q_{n_{2}},x,y)-f(x,y) \bigr\vert \\& \quad \leq M \Bigl\{ \tilde{\omega}_{2}\Bigl(f;\sqrt {A_{n_{1},n_{2}}^{(p_{1},p_{2})}(q_{n_{1}},q_{n_{2}},x,y)}\Bigr) +\min\bigl\{ 1,A_{n_{1},n_{2}}^{(p_{1},p_{2})}(q_{n_{1}},q_{n_{2}},x,y) \bigr\} \|f\|_{C(I_{1}\times I_{2})} \Bigr\} \\& \qquad {}+\omega \Bigl(f;\sqrt {B_{n_{1},n_{2}}^{(p_{1},p_{2})}(q_{n_{1}},q_{n_{2}},x,y)} \Bigr), \end{aligned}$$

*where*

$$\begin{aligned}& A_{n_{1},n_{2}}^{(p_{1},p_{2})}(q_{n_{1}},q_{n_{2}},x,y) \\& \quad = \biggl\{ \delta ^{2}_{n_{1}}(x)+\delta^{2}_{n_{2}}(y)+ \biggl(\frac{\alpha_{1}}{[n_{1}+1]_{q_{n_{1}}}+\beta_{1}}+\frac {2q_{n_{1}}[n_{1}]_{q_{n_{1}}}x+1}{[2]_{q_{n_{1}}}([n_{1}+1]_{q_{n_{1}}}+\beta _{1})}-x \biggr)^{2} \\& \qquad {}+ \biggl(\frac{\alpha_{2}}{[n_{2}+1]_{q_{n_{2}}}+\beta_{2}}+\frac {2q_{n_{2}}[n_{2}]_{q_{n_{2}}}y+1}{[2]_{q_{n_{2}}}([n_{2}+1]_{q_{n_{2}}}+\beta _{2})}-y \biggr)^{2} \biggr\} \end{aligned}$$

*and*

$$\begin{aligned} B_{n_{1},n_{2}}^{(p_{1},p_{2})}(q_{n_{1}},q_{n_{2}},x,y) =& \biggl(\frac{\alpha _{1}}{[n_{1}+1]_{q_{n_{1}}}+\beta_{1}}+\frac{2q_{n_{1}}[n_{1}]_{q_{n_{1}}} x+1}{[2]_{q_{n_{1}}}([n_{1}+1]_{q_{n_{1}}}+\beta_{1})}-x \biggr)^{2} \\ &{}+ \biggl(\frac{\alpha_{2}}{[n_{2}+1]_{q_{n_{2}}}+\beta_{2}}+\frac {2q_{n_{2}}[n_{2}]_{q_{n_{2}}}y+1}{[2]_{q_{n_{2}}}([n_{2}+1]_{q_{n_{2}}}+\beta _{2})}-y \biggr)^{2}, \end{aligned}$$

*and the constant* *M* (>0), *is independent of* *f* *and* $A_{n_{1},n_{2}}^{(p_{1},p_{2})}(q_{n_{1}},q_{n_{2}},x,y)$.

#### Proof

We define the auxiliary operators as follows:

3.4 $$\begin{aligned}& \mathcal{L}_{n_{1},n_{2},p_{1},p_{2}}^{(\alpha_{1},\alpha_{2},\beta_{1},\beta _{2})}(f;q_{n_{1}},q_{n_{2}},x,y) \\& \quad = \mathcal{K}_{n_{1},n_{2},p_{1},p_{2}}^{(\alpha _{1},\alpha_{2}, \beta_{1},\beta_{2})}(f;q_{n_{1}},q_{n_{2}},x,y) \\& \qquad {}-f \biggl(\frac{\alpha_{1}}{[n_{1}+1]_{q_{n_{1}}}+\beta_{1}}+\frac {2q_{n_{1}}[n_{1}]_{q_{n_{1}}}x+1}{[2]_{q_{n_{1}}}([n_{1}+1]_{q_{n_{1}}}+\beta _{1})}, \\& \qquad \frac{\alpha_{2}}{[n_{2}+1]_{q_{n_{2}}}+\beta_{2}}+\frac {2q_{n_{2}}[n_{2}]_{q_{n_{2}}}y+1}{[2]_{q_{n_{2}}}([n_{2}+1]_{q_{n_{2}}}+\beta _{2})} \biggr)+f(x,y). \end{aligned}$$

Considering Lemma 3, one has $\mathcal{L}_{n_{1},n_{2},p_{1},p_{2}}^{(\alpha _{1},\alpha_{2}, \beta_{1},\beta_{2})}(1;q_{n_{1}},q_{n_{2}},x,y)=1$, $\mathcal{L}_{n_{1},n_{2},p_{1},p_{2}}^{(\alpha_{1},\alpha_{2},\beta_{1},\beta _{2})}((t-x);q_{n_{1}},q_{n_{2}}, x,y)=0$, and $\mathcal {L}_{n_{1},n_{2},p_{1},p_{2}}^{(\alpha_{1},\alpha_{2},\beta_{1},\beta _{2})}((s-y);q_{n_{1}},q_{n_{2}},x,y)=0$.

Let $g\in C^{2}(I_{1}\times I_{2})$ and $(x,y)\in J^{2} $. Using Taylor’s theorem, we may write

$$\begin{aligned} g(t,s)-g(x,y) =&g(t,y)-g(x,y)+g(t,s)-g(t,y) \\ =&\frac{\partial g(x,y)}{\partial x}(t-x)+ \int^{t}_{x}(t-u)\frac{\partial ^{2} g(u,y)}{\partial u^{2}}\, du \\ &{}+\frac{\partial g(x,y)}{\partial y}(s-y)+ \int^{s}_{y}(s-v)\frac{\partial ^{2} g(x,v)}{\partial v^{2}}\, dv. \end{aligned}$$

Applying the operator $\mathcal{L}_{n_{1},n_{2},p_{1},p_{2}}^{(\alpha_{1},\alpha _{2},\beta_{1},\beta_{2})}(\cdot;q_{n_{1}},q_{n_{2}},x,y)$ on the above equation and using (3.4), we are led to

$$\begin{aligned}& \mathcal{L}_{n_{1},n_{2},p_{1},p_{2}}^{(\alpha_{1},\alpha_{2},\beta_{1},\beta _{2})}(g;q_{n_{1}},q_{n_{2}},x,y)-g(x,y) \\& \quad = \mathcal{L}_{n_{1},n_{2},p_{1},p_{2}}^{(\alpha_{1},\alpha_{2},\beta_{1},\beta _{2})} \biggl( \int^{t}_{x}(t-u)\frac{\partial^{2} g(u,y)}{\partial u^{2}} \,du;q_{n_{1}},q_{n_{2}},x,y \biggr) \\& \qquad {}+\mathcal{L}_{n_{1},n_{2},p_{1},p_{2}}^{(\alpha_{1},\alpha_{2},\beta_{1},\beta _{2})} \biggl( \int^{s}_{y}(s-v)\frac{\partial^{2} g(x,v)}{\partial v^{2}} \,dv;q_{n_{1}},q_{n_{2}},x,y \biggr) \\& \quad = \mathcal{K}_{n_{1},n_{2},p_{1},p_{2}}^{(\alpha_{1},\alpha_{2}, \beta_{1},\beta _{2})} \biggl( \int^{t}_{x}(t-u)\frac{\partial^{2} g(u,y)}{\partial u^{2}} \,du;q_{n_{1}},q_{n_{2}},x,y \biggr) \\& \qquad {}- \int_{x}^{\frac{\alpha_{1}}{[n_{1}+1]_{q_{1}}+\beta_{1}}+\frac {2q_{n_{1}}[n_{1}]_{q_{n_{1}}}x+1}{[2]_{q_{n_{1}}}([n_{1}+1]_{q_{n_{1}}}+\beta _{1})}} \biggl(\frac{\alpha_{1}}{[n_{1}+1]_{q_{n_{1}}}+\beta_{1}}+ \frac {2q_{n_{1}}[n_{1}]_{q_{n_{1}}}x+1}{[2]_{q_{n_{1}}}([n_{1}+1]_{q_{n_{1}}}+\beta _{1})}-u \biggr) \\& \qquad {}\times\frac{\partial^{2} g(u,y)}{\partial u^{2}}\,du+ \mathcal{K}_{n_{1},n_{2},p_{1},p_{2}}^{(\alpha_{1},\alpha_{2}, \beta_{1},\beta _{2})} \biggl( \int^{s}_{y}(s-v)\frac{\partial^{2} g(x,v)}{\partial v^{2}} \,dv;q_{n_{1}},q_{n_{2}},x,y \biggr) \\& \qquad {}- \int_{y}^{\frac{\alpha_{2}}{[n_{2}+1]_{q_{n_{2}}}+\beta_{2}}+\frac {2q_{n_{2}}[n_{2}]_{q_{n_{2}}}y+1}{[2]_{q_{n_{2}}}([n_{2}+1]_{q_{n_{2}}}+\beta _{2})}} \biggl(\frac{\alpha_{2}}{[n_{2}+1]_{q_{n_{2}}}+\beta_{2}}+ \frac {2q_{n_{2}}[n_{2}]_{q_{n_{2}}}y+1}{[2]_{q_{n_{2}}}([n_{2}+1]_{q_{n_{2}}}+\beta _{2})}-v \biggr) \\& \qquad {}\times\frac{\partial^{2} g(x,v)}{\partial v^{2}}\,dv. \end{aligned}$$

Hence,

3.5 $$\begin{aligned}& \bigl\vert \mathcal{L}_{n_{1},n_{2},p_{1},p_{2}}^{(\alpha_{1},\alpha_{2},\beta_{1},\beta _{2})}(g;q_{n_{1}},q_{n_{2}},x,y)-g(x,y) \bigr\vert \\& \quad \leq \mathcal{K}_{n_{1},n_{2},p_{1},p_{2}}^{(\alpha_{1},\alpha_{2}, \beta_{1},\beta _{2})} \biggl( \biggl\vert \int^{t}_{x} \bigl\vert (t-u) \bigr\vert \biggl\vert \frac{\partial^{2} g(u,y)}{\partial u^{2}} \biggr\vert \, du \biggr\vert ;q_{n_{1}},q_{n_{2}},x,y \biggr) \\& \qquad {} + \biggl\vert \int_{x}^{\frac{\alpha_{1}}{[n_{1}+1]_{q_{n_{1}}}+\beta_{1}}+\frac {2q_{n_{1}}[n_{1}]_{q_{n_{1}}}x+1}{[2]_{q_{n_{1}}}([n_{1}+1]_{q_{n_{1}}}+\beta _{1})}} \biggl\vert \biggl( \frac{\alpha_{1}}{[n_{1}+1]_{q_{n_{1}}}+\beta_{1}}+\frac {2q_{n_{1}}[n_{1}]_{q_{n_{1}}}x+1}{[2]_{q_{n_{1}}}([n_{1}+1]_{q_{n_{1}}}+\beta _{1})}-u \biggr) \biggr\vert \\& \qquad {}\times \biggl\vert \frac{\partial^{2} g(u,y)}{\partial u^{2}} \biggr\vert \, du \biggr\vert +\mathcal{K}_{n_{1},n_{2},p_{1},p_{2}}^{(\alpha_{1},\alpha_{2}, \beta_{1},\beta_{2})} \biggl( \biggl\vert \int^{s}_{y} \bigl\vert (s-v) \bigr\vert \biggl\vert \frac {\partial^{2} g(x,v)}{\partial v^{2}} \biggr\vert \, dv \biggr\vert ;q_{n_{1}},q_{n_{2}},x,y \biggr) \\& \qquad {}+ \biggl\vert \int_{y}^{\frac{\alpha_{2}}{[n_{2}+1]_{q_{n_{2}}}+\beta _{2}}+\frac {2q_{n_{2}}[n_{2}]_{q_{n_{2}}}y+1}{[2]_{q_{n_{2}}}([n_{2}+1]_{q_{n_{2}}}+\beta _{2})}} \biggl\vert \biggl( \frac{\alpha_{2}}{[n_{2}+1]_{q_{n_{2}}}+\beta_{2}}+\frac {2q_{n_{2}}[n_{2}]_{q_{n_{2}}}y+1}{[2]_{q_{n_{2}}}([n_{2}+1]_{q_{n_{2}}}+\beta _{2})}-v \biggr) \biggr\vert \\& \qquad {}\times\biggl\vert \frac{\partial^{2} g(x,v)}{\partial v^{2}} \biggr\vert \, dv \biggr\vert \\& \quad = A_{n_{1},n_{2}}^{p_{1},p_{2}}(q_{n_{1}},q_{n_{2}},x,y) \|g\|_{C^{2}(I_{1}\times I_{2})}. \end{aligned}$$

Also,

3.6 $$\begin{aligned}& \bigl\vert \mathcal{L}_{n_{1},n_{2},p_{1},p_{2}}^{(\alpha_{1},\alpha_{2},\beta_{1},\beta _{2})}(f;q_{n_{1}},q_{n_{2}},x,y) \bigr\vert \\& \quad \leq \bigl\vert \mathcal{K}_{n_{1},n_{2},p_{1},p_{2}}^{(\alpha_{1},\alpha_{2}, \beta_{1},\beta _{2})}(f;q_{n_{1}},q_{n_{2}},x,y) \bigr\vert +\biggl\vert f\biggl(\frac{\alpha _{1}}{[n_{1}+1]_{q_{n_{1}}}+\beta_{1}}+\frac {2q_{n_{1}}[n_{1}]_{q_{n_{1}}}x+1}{[2]_{q_{n_{1}}}([n_{1}+1]_{q_{n_{1}}}+\beta _{1})}, \\& \qquad {}\frac{\alpha_{2}}{[n_{2}+1]_{q_{n_{2}}}+\beta_{2}}+\frac {2q_{n_{2}}[n_{2}]_{q_{n_{2}}}y+1}{[2]_{q_{n_{2}}}([n_{2}+1]_{q_{n_{2}}}+\beta _{2})}\biggr)\biggr\vert +\bigl\vert f(x,y)\bigr\vert \\& \quad \leq 3\|f\|_{C(I_{1}\times I_{2})}. \end{aligned}$$

Hence, considering (3.4), (3.6), and (3.5) (in that order),

$$\begin{aligned}& \bigl\vert \mathcal{K}_{n_{1},n_{2},p_{1},p_{2}}^{(\alpha_{1},\alpha_{2},\beta _{1},\beta_{2})}(f;q_{n_{1}},q_{n_{2}},x,y)-f(x,y) \bigr\vert \\& \quad = \biggl\vert \mathcal{L}^{n_{1},n_{2},p_{1},p_{2}}_{(\alpha_{1},\alpha_{2},\beta_{1},\beta _{2})}(f;q_{n_{1}},q_{n_{2}},x,y)-f(x,y)+f \biggl(\frac{\alpha _{1}}{[n_{1}+1]_{q_{n_{1}}}+\beta_{1}}+\frac {2q_{n_{1}}[n_{1}]_{q_{n_{1}}}x+1}{[2]_{q_{n_{1}}}([n_{1}+1]_{q_{n_{1}}}+\beta _{1})}, \\& \qquad \frac{\alpha_{2}}{[n_{2}+1]_{q_{n_{2}}}+\beta_{2}}+\frac {2q_{n_{2}}[n_{2}]_{q_{n_{2}}}y+1}{[2]_{q_{n_{2}}}([n_{2}+1]_{q_{n_{2}}}+\beta _{2})} \biggr)-f(x,y) \biggr\vert \\& \quad \leq \bigl\vert \mathcal{L}^{n_{1},n_{2},p_{1},p_{2}}_{(\alpha_{1},\alpha_{2},\beta_{1},\beta _{2})}(f-g;q_{n_{1}},q_{n_{2}},x,y) \bigr\vert +\bigl\vert \mathcal{L}^{n_{1},n_{2},p_{1},p_{2}}_{(\alpha _{1},\alpha_{2},\beta_{1},\beta _{2})}(g;q_{n_{1}},q_{n_{2}},x,y)-g(x,y) \bigr\vert \\& \qquad {}+\bigl\vert g(x,y)-f(x,y)\bigr\vert \\& \qquad {}+ \biggl\vert f \biggl(\frac{\alpha_{1}}{[n_{1}+1]_{q_{n_{1}}}+\beta_{1}}+\frac {2q_{n_{1}}[n_{1}]_{q_{n_{1}}}x+1}{[2]_{q_{n_{1}}}([n_{1}+1]_{q_{n_{1}}}+\beta _{1})}, \\& \qquad \frac{\alpha_{2}}{[n_{2}+1]_{q_{n_{2}}}+\beta_{2}}+\frac {2q_{n_{2}}[n_{2}]_{q_{n_{2}}}y+1}{[2]_{q_{n_{2}}}([n_{2}+1]_{q_{n_{2}}}+\beta _{2})} \biggr)-f(x,y) \biggr\vert \\& \quad \leq 4\|f-g\|_{C(I_{1}\times I_{2})}+\bigl\vert \mathcal {K}^{n_{1},n_{2},p_{1},p_{2}}_{(\alpha_{1},\alpha_{2},\beta_{1},\beta _{2})}(g;q_{n_{1}},q_{n_{2}},x,y)-g(x,y) \bigr\vert \\& \qquad {} + \biggl\vert f \biggl(\frac{\alpha_{1}}{[n_{1}+1]_{q_{n_{1}}}+\beta_{1}}+\frac {2q_{n_{1}}[n_{1}]_{q_{n_{1}}}x+1}{[2]_{q_{n_{1}}}([n_{1}+1]_{q_{n_{1}}}+\beta_{1})}, \\& \qquad \frac{\alpha_{2}}{[n_{2}+1]_{q_{2}}+\beta_{2}}+\frac {2q_{2}[n_{2}]_{q_{2}}y+1}{[2]_{q_{2}}([n_{2}+1]_{q_{2}}+\beta_{2})} \biggr)-f(x,y) \biggr\vert \\& \quad \leq \bigl(4\|f-g\|_{C(I_{1}\times I_{2})}+A_{n_{1},n_{2}}^{p_{1},p_{2}}(q_{n_{1}},q_{n_{2}},x,y) \|g\|_{C^{2}(I_{1}\times I_{2})} \bigr) \\& \qquad {}+\omega \Bigl(f;\sqrt {B_{n_{1},n_{2}}^{(p_{1},p_{2})}(q_{n_{1}},q_{n_{2}},x,y)} \Bigr). \end{aligned}$$

Now, taking the infimum on the right hand side all over $g\in C^{2}(I_{1}\times I_{2})$ and using (3.3)

$$\begin{aligned}& \bigl\vert \mathcal{K}_{n_{1},n_{2},p_{1},p_{2}}^{(\alpha_{1},\alpha_{2},\beta_{1},\beta _{2})}(f;q_{n_{1}},q_{n_{2}},x,y)-f(x,y) \bigr\vert \\& \quad \leq 4\mathcal {K}\bigl(f;A_{n_{1},n_{2}}^{(p_{1},p_{2})}(q_{n_{1}},q_{n_{2}},x,y) \bigr) +\omega \Bigl(f;\sqrt{B_{n_{1},n_{2}}^{(p_{1},p_{2})}(q_{n_{1}},q_{n_{2}},x,y)} \Bigr) \\& \quad \leq M \Bigl\{ \tilde{\omega}_{2} \Bigl(f;\sqrt {A_{n_{1},n_{2}}^{p_{1},p_{2}}(q_{n_{1}},q_{n_{2}},x,y)} \Bigr) + \min\bigl\{ 1,A_{n_{1},n_{2}}^{(p_{1},p_{2})}(q_{n_{1}},q_{n_{2}},x,y) \bigr\} \|f\|_{C(I_{1}\times I_{2})} \Bigr\} \\& \qquad {}+\omega \Bigl(f;\sqrt {B_{n_{1},n_{2}}^{(p_{1},p_{2})}(q_{n_{1}},q_{n_{2}},x,y)} \Bigr). \end{aligned}$$

Thus, we get the desired result. □

#### Theorem 8

*Let* $f\in C^{2}(I_{1}\times I_{2})$. *Then for every* $(x,y)\in J^{2}$,

$$\begin{aligned}& \lim_{[n]_{q_{n}}\rightarrow\infty}[n]_{q_{n}}\bigl\{ \mathcal {K}_{n,n,p_{1},p_{2}}^{(\alpha_{1},\alpha_{2},\beta_{1},\beta _{2})}\bigl(f(t,s);q_{n},x,y\bigr)-f(x,y) \bigr\} \\& \quad = f_{x}(x,y) \biggl(\frac{-x(a+1+2\beta _{1})}{2}+\alpha_{1}+ \frac{1}{2} \biggr) +f_{y}(x,y) \biggl(\frac{-y(a+1+2\beta _{2})}{2}+\alpha_{2}+ \frac{1}{2} \biggr) \\& \qquad {}+\frac{1}{2}\biggl\{ f_{xx}(x,y)\frac {x(1-x)}{2}+f_{yy}(x,y) \frac{y(1-y)}{2}\biggr\} \end{aligned}$$

*uniformly in* $(x,y)\in J^{2}$.

#### Proof

By Taylor’s formula for *f*, we have

$$\begin{aligned} f(t,s) =&f(x,y)+f_{x}(x,y) (t-x)+f_{y}(x,y) (s-y) \\ &{}+ \frac{1}{2}\bigl\{ f_{xx}(x,y) (t-x)^{2} +2f_{xy}(x,y) (t-x) (s-y)+f_{yy}(x,y) (s-y)^{2}\bigr\} \\ &{}+\xi (t,s,x,y)\sqrt{(t-x)^{4}+(s-y)^{4}}, \end{aligned}$$

where $\xi(t,s,x,y)\rightarrow0$ as $(t,s)\rightarrow(x,y)$ and $\xi (t,s,x,y)\in C^{2}(I_{1}\times I_{2})$. Now, applying the operator $\mathcal{K}_{n,n,p_{1},p_{2}}^{(\alpha_{1},\alpha _{2}, \beta_{1},\beta_{2})}(\cdot;q_{n},x,y)$ on the above equation, we get

$$\begin{aligned}& \mathcal{K}_{n,n,p_{1},p_{2}}^{(\alpha_{1},\alpha_{2}, \beta_{1},\beta _{2})}\bigl(f(t,s);q_{n},x,y \bigr) \\& \quad = f(x,y)+f_{x}(x,y)\mathcal{K}_{n,p_{1}}^{(\alpha _{1},\beta_{1})} \bigl((t-x);q_{n},x\bigr)+f_{y}(x,y)\mathcal{K}_{n,p_{2}}^{(\alpha_{2},\beta _{2})} \bigl((s-y);q_{n},y\bigr) \\& \qquad {}+\frac{1}{2}\bigl\{ f_{xx}(x,y)\mathcal {K}_{n,p_{1}}^{(\alpha_{1},\beta_{1})}\bigl((t-x)^{2};q_{n},x \bigr)+2f_{xy}(x,y)\mathcal {K}_{n,p_{1}}^{(\alpha_{1},\beta_{1})} \bigl((t-x);q_{n},x\bigr) \\& \qquad {}\times\mathcal {K}_{n,p_{2}}^{\alpha_{2},\beta_{2}}\bigl((s-y);q_{n},y \bigr)+f_{yy}\mathcal {K}_{n,p_{2}}^{\alpha_{2},\beta_{2}} \bigl((s-y)^{2};q_{n},y\bigr)\bigr\} \\& \qquad {}+\mathcal {K}_{n,n,p_{1},p_{2}}^{(\alpha_{1},\alpha_{2},\beta_{1},\beta_{2})}\bigl(\xi (t,s,x,y) \sqrt{(t-x)^{4}+(s-y)^{4}};q_{n},x,y\bigr). \end{aligned}$$

Hence, using Lemma 2,

$$\begin{aligned}& \lim_{[n]_{q_{n}}\rightarrow\infty}[n]_{q_{n}}\bigl\{ \mathcal {K}_{n,n,p_{1},p_{2}}^{(\alpha_{1},\alpha_{2},\beta_{1},\beta _{2})}\bigl(f(t,s);q_{n},x,y\bigr)-f(x,y) \bigr\} \\& \quad = f_{x}(x,y) \biggl(\frac{-x(a+1+2\beta _{1})}{2}+\alpha_{1}+ \frac{1}{2} \biggr) +f_{y}(x,y) \biggl(\frac{-y(a+1+2\beta _{2})}{2}+\alpha_{2}+ \frac{1}{2} \biggr) \\& \qquad {}+\frac{1}{2}\biggl\{ f_{xx}(x,y)\frac {x(1-x)}{2}+f_{yy}(x,y) \frac{y(1-y)}{2}\biggr\} \\& \qquad {}+\lim_{[n]_{q_{n}}\rightarrow\infty}[n]_{q_{n}}\mathcal {K}_{n,n,p_{1},p_{2}}^{(\alpha_{1},\alpha_{2},\beta_{1},\beta_{2})} \bigl(\xi (t,s,x,y)\sqrt{(t-x)^{4}+(s-y)^{4}};x,y \bigr) \end{aligned}$$

uniformly in $(x,y)\in J^{2}$.

Applying the Cauchy-Schwarz inequality

$$\begin{aligned}& \bigl\vert \mathcal{K}_{n,n,p_{1},p_{2}}^{(\alpha_{1},\alpha_{2},\beta_{1},\beta_{2})}\bigl(\xi (t,s) \sqrt{(t-x)^{4}+(s-y)^{4}};q_{n},x,y\bigr)\bigr\vert \\& \quad \leq\sqrt{\mathcal{K}_{n,n,p_{1},p_{2}}^{\alpha_{1},\alpha_{2},\beta_{1},\beta _{2}}\bigl( \xi^{2}(t,s);q_{n},x,y\bigr)}\sqrt{ \mathcal{K}_{n,n,p_{1},p_{2}}^{(\alpha _{1},\alpha_{2},\beta_{1},\beta_{2})}\bigl((t-x)^{4}+(s-y)^{4};q_{n},x,y \bigr)}. \end{aligned}$$

Since, by Theorem 2 and in view of Lemma 2,

$$\begin{aligned}& \lim_{[n]_{q_{n}}\rightarrow\infty}\mathcal{K}_{n,n,p_{1},p_{2}}^{(\alpha _{1},\alpha_{2},\beta_{1},\beta_{2})}\bigl( \xi^{2}(t,s);x,y\bigr)=\xi^{2}(x,y)=0, \\& \mathcal{K}_{n,n,p_{1},p_{2}}^{(\alpha_{1},\alpha_{2},\beta_{1},\beta _{2})}\bigl((t-x)^{4};q_{n},x \bigr)=O \biggl(\frac{1}{[n]_{q_{n}}^{2}} \biggr),\quad \mbox{and} \\& \mathcal{K}_{n,p_{2}}^{(\alpha_{1},\alpha_{2},\beta_{1},\beta _{2})}\bigl((s-y)^{4};q_{n},y \bigr)=O \biggl(\frac{1}{[n]_{q_{n}}^{2}} \biggr) \end{aligned}$$

uniformly in $(x,y)\in J^{2}$, it follows that

$$\lim_{n\rightarrow\infty}[n]_{q_{n}}\bigl\{ \mathcal {K}_{n,n,p_{1},p_{2}}^{(\alpha_{1},\alpha_{2},\beta_{1},\beta_{2})}\bigl(\xi(t,s)\sqrt {(t-x)^{4}+(s-y)^{4}};q_{n},x,y \bigr)\bigr\} =0 $$

uniformly in $(x,y)\in J^{2}$, the desired result is obtained. □

In the following example, the rate of convergence of the bivariate operators given by (3.1) to a certain function is shown by illustrative graphics. We observe that when the values of $q_{1}$ and $q_{2}$ increase, the approximation of *f* by the operator $\mathcal {K}_{n_{1},n_{2},p_{1}p_{2}}^{ ( \alpha_{1},\alpha_{2},\beta _{1},\beta _{2} ) } ( f;q_{1},q_{2},x,y ) $ becomes better.

#### Example 3

**Figure 4:**
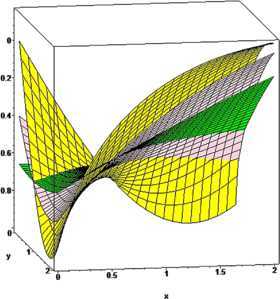
**The convergence of** $\pmb{K_{n_{1},n_{2},p_{1},p_{2}}^{(\alpha_{1},\alpha_{2},\beta_{1},\beta_{2})}(f;q_{1},q_{2},x,y)}$ **to** $\pmb{f(x,y)}$ **.**

Let $n_{1}=n_{2}=5$, $\alpha_{1}=0.5$, $\beta_{1}=0.6$, $\alpha _{2}=0.7$, $\beta_{2}=0.8$, $p_{1}=p_{2}=1$. For $q_{1}=0.45$, $q_{2}=0.50$ (green) and $q_{1}=0.85$, $q_{2}=0.90$ (pink), the convergence of the operators $\mathcal {K}_{n_{1},n_{2},p_{1}p_{2}}^{ ( \alpha_{1},\alpha_{2},\beta _{1},\beta _{2} ) } ( f;q_{1},q_{2},x,y ) $ given by (3.1) to $f ( x,y ) =\sin ( x+y ) /(1+xy)$ (yellow) is illustrated in Figure 4.

## Construction of GBS operator of *q*-Bernstein-Schurer-Kantorovich type

In [7] and [8], Bögel proposed the concepts of *B*-continuous and *B*-differentiable functions. Later, Dobrescu and Matei [9] discussed the approximation of *B*-continuous functions on a bounded interval by a generalized Boolean sum of bivariate generalization of Bernstein polynomials. Subsequently, Badea and Cottin [10] established Korovkin theorems for GBS operators. Pop [11] studied the GBS operators associated to a certain class of linear and positive operators defined by an infinite sum and discussed the approximation of *B*-continuous and *B*-differentiable functions by these operators. Recently, Sidharth *et al.* [12] proposed the GBS operators of *q*-Bernstein-Schurer-Kantorovich type and studied the rate of convergence of these operators by means of the mixed modulus of smoothness. Agrawal and Ispir [13] introduced the bivariate generalization of Chlodowsky-Szasz-Charlier-type operators and obtained the degree of approximation for the associated GBS operators. In this section, we give some basic definitions and notations, for further details, one can see [14].

Let *X* and *Y* be compact subsets of $\mathbb{R}$. A function $f:X\times Y\longrightarrow \mathbb{R}$ is called a *B*-continuous (Bögel continuous) function at $(x_{0},y_{0})\in X\times Y$ if

$$\lim_{(x,y)\rightarrow(x_{0},y_{0})}\Delta f\bigl[(x_{0},y_{0});(x,y) \bigr]=0, $$

where $\Delta f[(x_{0},y_{0});(x,y)]$ denotes the mixed difference defined by

4.1 $$ \Delta f\bigl[(x_{0},y_{0});(x,y) \bigr]=f(x,y)-f(x,y_{0})-f(x_{0},y)+f(x_{0},y_{0}). $$

The function $f:X\times Y\rightarrow\mathbb{R}$ is called *B*-bounded on $X\times Y$ if there exists $M>0$ such that $\vert \Delta f [(t,s);(x,y) ]\vert \leq M$, for every $(x,y),(t,s)\in(X\times Y)$. Since $X\times Y$ is a compact subset of $\mathbb{R}^{2}$, each *B*-continuous function is a *B*-bounded function on $X\times Y\rightarrow\mathbb{R}$.

Throughout this paper, $B_{b} ( X\times Y )$ denotes all *B*-bounded functions on $X\times Y\rightarrow\mathbb{R}$, equipped with the norm $\Vert f\Vert _{B}=\sup_{ (x,y ), (t,s )\in X\times Y}\vert \Delta f [ (t,s);(x,y) ] \vert $. We denote by $C_{b} ( X\times Y )$, the space of all *B*-continuous functions on $X\times Y$. $B ( X\times Y ),C ( X\times Y ) $ denote the space of all bounded functions and the space of all continuous (in the usual sense) functions on $X\times Y$ endowed with the sup-norm $\Vert \cdot \Vert _{\infty}$. It is well known that $C (X\times Y ) \subset C_{b} ( X\times Y ) $ ([14], p.52).

A function $f: X\times Y\longrightarrow\mathbb{R}$ is called a *B*-differentiable (Bögel differentiable) function at $(x_{0},y_{0})\in X\times Y$ if the limit

$$\lim_{(x,y)\rightarrow(x_{0},y_{0})}\frac{\Delta f[(x_{0},y_{0});(x,y)]}{(x-x_{0})(y-y_{0})} $$

exists and is finite.

The limit is said to be the *B*-differential of *f* at the point $(x_{0},y_{0})$ and is denoted by $D_{B}(f;x_{0},y_{0})$ and the space of all *B*-differentiable functions is denoted by $D_{b}(X\times Y)$. The mixed modulus of smoothness of $f\in C_{b} (I_{1}\times I_{2} ) $ is defined as

$$ \omega_{\mathrm{mixed}} ( f;\delta_{1},\delta_{2} ) :=\sup \bigl\{ \bigl\vert \Delta f \bigl[ (t,s);(x,y) \bigr] \bigr\vert :\vert x-t \vert < \delta_{1},\vert y-s\vert < \delta_{2} \bigr\} $$

for all $(x,y), (t,s)\in(I_{1}\times I_{2})$ and for any $(\delta _{1},\delta_{2}) \in(0,\infty) \times(0,\infty) $ with $\omega _{\mathrm{mixed}}: [0,\infty ) \times [0,\infty ) \rightarrow\mathbb{R}$. The basic properties of $\omega_{\mathrm{mixed}}$ were obtained by Badea *et al.* in [15] and [16], which are similar to the properties of the usual modulus of continuity.

We define the GBS operator of the operator $\mathcal {K}_{n_{1},n_{2},p_{1},p_{2}}^{(\alpha_{1},\alpha_{2}, \beta_{1},\beta_{2})}$ given by (1.3), for any $f\in C_{b} ( I_{1}\times I_{2} ) $ and $m,n\in\mathbb{N}$, by

4.2 $$\begin{aligned}& T_{n_{1},n_{2},p_{1},p_{2}}^{(\alpha_{1},\alpha_{2},\beta_{1},\beta _{2})}\bigl(f(t,s);q_{n_{1}},q_{n_{2}},x,y \bigr) \\& \quad :=\mathcal{K}_{n_{1},n_{2},p_{1},p_{2}}^{(\alpha_{1},\alpha_{2},\beta_{1},\beta _{2})} \bigl(f ( t,y )+f (x,s ) -f (t,s );q_{n_{1}},q_{n_{2}},x,y \bigr) \end{aligned}$$

for all $(x,y )\in J^{2}$.

Hence for any $f\in C_{b} (I_{1}\times I_{2} )$, the GBS operator of the *q*-Bernstein-Schurer-Kantorovich type is

$$ T_{n_{1},n_{2},p_{1},p_{2}}^{(\alpha_{1},\alpha_{2},\beta_{1},\beta_{2})}: C_{b} (I_{1}\times I_{2} )\longrightarrow C(I_{1}\times I_{2}) $$

given by

$$\begin{aligned}& T_{n_{1},n_{2},p_{1},p_{2}}^{(\alpha_{1},\alpha_{2},\beta_{1},\beta _{2})}(f;q_{n_{1}},q_{n_{2}},x,y) \\& \quad = \sum_{k_{1}=0}^{n_{1}+p_{1}}\sum _{k_{2}=0}^{n_{2}+p_{2}} \tilde{p}_{n_{1},n_{2},k_{1},k_{2}}^{*}(q_{n_{1}},q_{n_{2}};x,y) \\& \qquad {}\times \int _{0}^{1} \int_{0}^{1}f \biggl\{ \biggl(\frac{[k_{1}]_{q_{n_{1}}}+q_{n_{1}}^{k_{1}}t+ \alpha_{1}}{[n_{1}+1]_{q_{n_{1}}}+\beta_{1}},y \biggr) +f \biggl(x,\frac {[k_{2}]_{q_{n_{2}}}+q_{n_{2}}^{k_{2}}s+\alpha_{2}}{[n_{2}+1]_{q_{n_{2}}}+\beta _{2}} \biggr) \\& \qquad {}-f \biggl(\frac{[k_{1}]_{q{n_{1}}}+q_{n_{1}}^{k_{1}}t+\alpha _{1}}{[n_{1}+1]_{q_{n_{1}}}+\beta_{1}},\frac {[k_{2}]_{q{n_{2}}}+q_{n_{2}}^{k_{2}}s+\alpha_{2}}{[n_{2}+1]_{q{n_{2}}}+\beta_{2}} \biggr) \biggr\} \, d_{q_{n_{1}}}t \, d_{q_{n_{2}}}s, \end{aligned}$$

where

$$\begin{aligned} \tilde{p}_{n_{1},n_{2},k_{1},k_{2}}^{*}(q_{n_{1}},q_{n_{2}},x,y) =& \frac {[n_{1}]_{q_{n_{1}}}^{n_{1}+p_{1}}}{[n_{1}+p_{1}]_{q_{n_{1}}}^{n_{1}+p_{1}}}{n_{1}+p_{1} \brack k_{1}}_{q_{n_{1}}} x^{k_{1}} \biggl(\frac{[n_{1}+p_{1}]_{q_{n_{1}}}}{[n_{1}]_{q_{n_{1}}}}-x \biggr)_{q_{n_{1}}}^{n_{1}+p_{1}-k_{1}} \\ &{}\times\frac {[n_{2}]_{q_{n_{2}}}^{n_{2}+p_{2}}}{[n_{2}+p_{2}]_{q_{n_{2}}}^{n_{2}+p_{2}}}{n_{2}+p_{2} \brack k_{2}}_{q_{n_{2}}} y^{k_{2}} \biggl(\frac{[n_{2}+p_{2}]_{q_{n_{2}}}}{[n_{2}]_{q_{n_{2}}}}-y \biggr)_{q_{n_{2}}}^{n_{2}+p_{2}-k_{2}}. \end{aligned}$$

Clearly, the operator $T_{n_{1},n_{2},p_{1},p_{2}}^{(\alpha_{1},\alpha_{2},\beta _{1},\beta_{2})}$ is linear and preserves linear functions.

### Theorem 9

*For every* $f\in C_{b} (I_{1}\times I_{2} )$, *at each point* $(x,y)\in J^{2}$, *the operator* (4.2) *verifies the following inequality*:

$$ \bigl\vert T_{n_{1},n_{2},p_{1},p_{2}}^{(\alpha_{1},\alpha_{2},\beta_{1},\beta _{2})}(f;q_{n_{1}},q_{n_{2}},x,y)-f(x,y) \bigr\vert \leq4 \omega_{\mathrm{mixed}}\bigl(f;\sqrt{\delta _{n_{1}}(x)}, \sqrt{\delta_{n_{2}}(y)}\bigr). $$

### Proof

By the property

$$ \omega_{\mathrm{mixed}}(f;\lambda_{1}\delta_{1}, \lambda_{2}\delta_{2})\leq (1+\lambda_{1}) (1+ \lambda_{2}) \omega_{\mathrm{mixed}}(f,\delta_{1}, \delta_{2});\quad \lambda_{1},\lambda_{2}>0, $$

we can write

4.3 $$\begin{aligned} \bigl\vert \Delta f\bigl[(t,s);(x,y)\bigr]\bigr\vert \leq& \omega_{\mathrm{mixed}}\bigl(f;\vert t-x\vert ,\vert s-y\vert \bigr) \\ \leq& \biggl(1+\frac{\vert t-x\vert }{\delta_{1}} \biggr) \biggl(1+\frac{\vert s-y\vert }{\delta _{2}} \biggr) \omega_{\mathrm{mixed}}(f;\delta_{1},\delta_{2}) \end{aligned}$$

for every $(t,s)\in(I_{1}\times I_{2})$, $(x,y)\in J^{2} $ and any $\delta _{1},\delta_{2}>0$. From (4.2) and the definition of the mixed difference $\Delta f[(t,s);(x,y)]$, on applying Lemma 3 and the inequality (4.3), we get

$$\begin{aligned}& \bigl\vert T_{n_{1},n_{2},p_{1},p_{2}}^{(\alpha_{1},\alpha_{2},\beta_{1},\beta _{2})}(f;q_{n_{1}},q_{n_{2}},x,y)-f(x,y) \bigr\vert \\& \quad \leq \mathcal{K}_{n_{1},n_{2},p_{1},p_{2}}^{(\alpha_{1},\alpha_{2}, \beta _{1},\beta_{2})} \bigl(\bigl\vert \Delta f\bigl[(t,s);(x,y)\bigr]\bigr\vert ;q_{n_{1}},q_{n_{2}},x,y \bigr) \\& \quad \leq \biggl(\mathcal{K}_{n_{1},p_{1}}^{(\alpha_{1}, \beta _{1})}(1;q_{n_{1}},x)+ \frac{1}{\sqrt{\delta_{n_{1}}}}\mathcal {K}_{n_{1},p_{1}}^{(\alpha_{1},\beta_{1})}\bigl(\vert t-x \vert ;q_{n_{1}},x\bigr) \\& \qquad {} +\frac{1}{\sqrt{\delta_{n_{2}}}}\mathcal{K}_{n_{2},p_{2}}^{(\alpha_{2},\beta _{2})}\bigl( \vert s-y\vert ;q_{n_{2}},y\bigr)+\frac{1}{\sqrt{\delta_{n_{1}}}\sqrt{\delta _{n_{2}}}} \mathcal{K}_{n_{1},p_{1}}^{(\alpha_{1},\beta_{1})}\bigl(\vert t-x\vert ;q_{n_{1}},x\bigr) \\& \qquad {} \times \mathcal{K}_{n_{2},p_{2}}^{(\alpha_{2},\beta _{2})}\bigl(\vert s-y \vert ;q_{n_{2}},y\bigr) \biggr)\omega_{\mathrm{mixed}}(f;\sqrt{ \delta_{n_{1}}},\sqrt {\delta_{n_{2}}}). \end{aligned}$$

Now, applying the Cauchy-Schwarz inequality

$$\begin{aligned}& \bigl\vert T_{n_{1},n_{2},p_{1},p_{2}}^{(\alpha_{1},\alpha_{2},\beta_{1},\beta _{2})}(f;q_{n_{1}},q_{n_{2}},x,y)-f(x,y) \bigr\vert \\& \quad \leq \biggl(\mathcal {K}_{n_{1},p_{1}}^{(\alpha_{1},\beta_{1})}(e_{00};q_{n_{1}},x)+ \frac{1}{\sqrt {\delta_{n_{1}}}}\sqrt{\mathcal{K}_{n_{1},p_{1}}^{(\alpha_{1},\beta _{1})} \bigl((t-x)^{2};q_{n_{1}},x\bigr)} \\& \qquad {}+\frac{1}{\sqrt{\delta_{n_{2}}}}\sqrt{\mathcal{K}_{n_{2},p_{2}}^{(\alpha _{2},\beta_{2})} \bigl((s-y)^{2};q_{n_{2}},y\bigr)} +\frac{1}{\sqrt{\delta_{n_{1}}}\sqrt{\delta_{n_{2}}}}\sqrt{\mathcal {K}_{n_{1},p_{1}}^{(\alpha_{1},\beta_{1})} \bigl((t-x)^{2};q_{n_{1}},x\bigr)} \\& \qquad {}\times\sqrt{\mathcal{K}_{n_{2},p_{2}}^{(\alpha_{2},\beta _{2})} \bigl((s-y)^{2};q_{n_{2}},y\bigr)} \biggr) \omega_{\mathrm{mixed}}(f, \delta_{n_{1}},\delta _{n_{2}}) \\& \quad = 4 \omega_{\mathrm{mixed}}(f;\sqrt{\delta_{n_{1}}},\sqrt{ \delta_{n_{2}}}), \end{aligned}$$

on choosing $\delta_{n_{1}}=\delta_{n_{1}}(x)$ and $\delta_{n_{2}}=\delta _{n_{2}}(y)$. This completes the proof. □

Next, let us define the Lipschitz class for *B*-continuous functions. For $f\in C_{b} (I_{1}\times I_{2} )$, the Lipschitz class $\operatorname{Lip}_{M} (\xi,\eta )$ with $\xi,\eta\in ( 0,1 ] $ is defined by

$$\begin{aligned} \operatorname{Lip}_{M} ( \xi,\eta ) =& \bigl\{ f\in C_{b} ( I_{1}\times I_{2} ) :\bigl\vert \Delta f \bigl[ (t,s);(x,y) \bigr] \bigr\vert \leq M\vert t-x\vert ^{\xi} \vert s-y\vert ^{\eta }, \\ &\text{for } (t,s ), (x,y ) \in I_{1}\times I_{2} \bigr\} . \end{aligned}$$

In our next result, we determine the degree of approximation for the operators $T_{n_{1},n_{2},p_{1},p_{2}}^{(\alpha_{1},\beta_{1},\alpha_{2},\beta_{2})}$ by means of the class $\operatorname{Lip}_{M}(\xi,\eta)$ of the class of Bögel continuous functions.

### Theorem 10

*For* $f\in \operatorname{Lip}_{M} (\xi,\eta )$, *we have*

$$ \bigl\vert T_{n_{1},n_{2},p_{1},p_{2}}^{(\alpha_{1},\alpha_{2},\beta_{1},\beta _{2})}(f;q_{n_{1}},q_{n_{2}},x,y)-f(x,y) \bigr\vert \leq M \bigl(\delta_{n_{1}}(x)\bigr)^{\frac{\xi }{2}}\bigl( \delta_{n_{2}}(y)\bigr)^{\frac{\eta}{2}} $$

*for* $M>0$, $\xi,\eta\in(0,1]$.

### Proof

From (4.2), (4.1), and by our hypothesis, we may write

$$\begin{aligned}& \bigl\vert T_{n_{1},n_{2},p_{1},p_{2}}^{(\alpha_{1},\alpha_{2},\beta_{1},\beta _{2})} ( f;q_{n_{1}},q_{n_{2}},x,y ) -f ( x,y ) \bigr\vert \\& \quad \leq \mathcal{K}_{n_{1},n_{2},p_{1},p_{2}}^{(\alpha _{1},\alpha_{2},\beta_{1},\beta_{2})} \bigl( \bigl\vert \Delta f \bigl[ (t,s);(x,y) \bigr] \bigr\vert ;x,y \bigr) \\& \quad \leq M \mathcal{K}_{n_{1},n_{2},p_{1},p_{2}}^{(\alpha_{1},\alpha_{2},\beta_{1},\beta _{2})} \bigl( \vert t-x\vert ^{\xi }\vert s-y\vert ^{\eta};x,y \bigr) \\& \quad = M \mathcal{K}_{n_{1},p_{1}}^{(\alpha_{1},\beta_{1})} \bigl( \vert t-x\vert ^{\xi};x \bigr) \overline{K}_{n_{2},p_{2}}^{(\alpha_{2},\beta_{2})} \bigl( \vert s-y \vert ^{\eta};y \bigr) . \end{aligned}$$

Applying Hölder’s inequality with $p_{1}=2/\xi$, $q_{1}=2/ ( 2-\xi ) $ and $p_{2}=2/\eta$, $q_{2}=2/ ( 2-\eta ) $, we are led to

$$\begin{aligned}& \bigl\vert T_{n_{1},n_{2},p_{1},p_{2}}^{(\alpha_{1},\beta_{1},\alpha_{2},\beta _{2})} ( f;q_{n_{1}},q_{n_{2}},x,y ) -f ( x,y ) \bigr\vert \\& \quad \leq M \bigl( \mathcal{K}_{n_{1},p_{1}}^{(\alpha _{1},\beta_{1})} \bigl((t-x)^{2};x\bigr) \bigr) ^{\xi/2}\mathcal{K}_{n_{1},p_{1}}^{(\alpha_{1},\beta_{1})} ( e_{0};x ) ^{(2-\xi)/2} \\& \qquad {}\times \bigl(\mathcal{K}_{n_{2},p_{2}}^{(\alpha_{2},\beta _{2})}\bigl((s-y)^{2};y \bigr) \bigr) ^{\eta /2}\mathcal{K}_{n_{2},p_{2}}^{(\alpha_{2},\beta_{2})} ( e_{0};y ) ^{(2-\eta)/2}. \end{aligned}$$

In view of Lemma 1, the desired result is immediate. □

### Theorem 11

*For* $f\in D_{b}(I_{1}\times I_{2})$ *with* $D_{B}f\in B(I_{1}\times I_{2})$ *and each* $(x,y)\in J^{2}$, *we have*

$$\begin{aligned}& \bigl\vert T_{n_{1},n_{2},p_{1},p_{2}}^{(\alpha_{1},\beta_{1},\alpha_{2},\beta _{2})}(f;q_{n_{1}},q_{n_{2}},x,y)-f(x,y) \bigr\vert \\& \quad \leq \frac {M}{[n_{1}]_{q_{n_{1}}}^{1/2}[n_{2}]_{q_{n_{2}}}^{1/2}} \bigl(\Vert D_{B} f\Vert _{\infty} +\omega_{\mathrm{mixed}}\bigl(D_{B} f;[n_{1}]_{q_{n_{1}}}^{-1/2},[n_{2}]_{q_{n_{2}}}^{-1/2} \bigr) \bigr). \end{aligned}$$

### Proof

By our hypothesis, using the relations

$$ \Delta f\bigl[(t,s);(x,y)\bigr]=(t-x) (s-y)D_{B} f(\xi,\eta), \quad \mbox{where } x< \xi< t ; y< \eta< s, $$

and

$$ D_{B} f(\xi,\eta)=\Delta D_{B} f(\xi, \eta)+D_{B} f(\xi,y)+D_{B} f(x,\eta)-D_{B} f(x,y), $$

we obtain

$$\begin{aligned}& \bigl\vert \mathcal{K}_{n_{1},n_{2},p_{1},p_{2}}^{(\alpha_{1},\alpha_{2},\beta_{1},\beta _{2})}\bigl(\Delta f \bigl[(t,s);(x,y)\bigr];q_{n_{1}},q_{n_{2}},x,y\bigr)\bigr\vert \\& \quad = \bigl\vert \mathcal {K}_{n_{1},n_{2},p_{1},p_{2}}^{(\alpha_{1},\alpha_{2},\beta_{1},\beta _{2})}\bigl((t-x) (s-y)D_{B} f(\xi,\eta);q_{n_{1}},q_{n_{2}},x,y\bigr) \bigr\vert \\& \quad \leq \mathcal{K}_{n_{1},n_{2},p_{1},p_{2}}^{(\alpha_{1},\alpha_{2},\beta_{1},\beta _{2})}\bigl(\vert t-x\vert \vert s-y\vert \bigl\vert \Delta D_{B} f(\xi,\eta)\bigr\vert ;x,y\bigr) \\& \qquad {}+\mathcal{K}_{n_{1},n_{2},p_{1},p_{2}}^{(\alpha_{1},\alpha_{2},\beta_{1},\beta _{2})}\bigl(\vert t-x\vert \vert s-y\vert \bigl(\bigl\vert D_{B} f(\xi,y)\bigr\vert \\& \qquad {}+\bigl\vert D_{B} f(x,\eta)\bigr\vert +\bigl\vert D_{B} f(x,y)\bigr\vert \bigr);q_{n_{1}},q_{n_{2}},x,y \bigr) \\& \quad \leq \mathcal{K}_{n_{1},n_{2},p_{1},p_{2}}^{(\alpha_{1},\alpha_{2},\beta_{1},\beta _{2})}\bigl(\vert t-x\vert \vert s-y\vert \omega_{\mathrm{mixed}}\bigl(D_{B} f;\vert \xi-x \vert ,\vert \eta-y\vert \bigr);x,y\bigr) \\& \qquad {}+3 \|D_{B} f\|_{\infty} \mathcal{K}_{n_{1},n_{2},p_{1},p_{2}}^{(\alpha _{1},\alpha_{2},\beta_{1},\beta_{2})} \bigl(\vert t-x\vert \vert s-y\vert ;q_{n_{1}},q_{n_{2}},x,y \bigr). \end{aligned}$$

Hence taking into account and applying the Cauchy-Schwarz inequality we obtain

$$ \omega_{\mathrm{mixed}}\bigl(D_{B} f;\vert \xi-x\vert ,\vert \eta-y\vert \bigr)\leq \biggl(1+\frac{|t-x|}{\delta _{n_{1}}} \biggr) \biggl(1+ \frac{|s-y|}{\delta_{n_{2}}} \biggr)\omega_{\mathrm{mixed}}(D_{B} f; \delta_{n_{1}},\delta_{n_{2}}). $$

We have

4.4 $$\begin{aligned}& \bigl\vert T_{n_{1},n_{2},p_{1},p_{2}}^{(\alpha_{1},\alpha_{2},\beta_{1},\beta _{2})}(f;q_{n_{1}},q_{n_{2}},x,y)-f(x,y) \bigr\vert \\& \quad \leq 3\|D_{B} f\|_{\infty}\sqrt{ \mathcal{K}_{n_{1},n_{2},p_{1},p_{2}}^{(\alpha _{1},\alpha_{2},\beta_{1},\beta _{2})}\bigl((t-x)^{2}(s-y)^{2};q_{n_{1}},q_{n_{2}},x,y \bigr)} \\& \qquad {} + \Bigl(\sqrt{\mathcal{K}_{n_{1},n_{2},p_{1},p_{2}}^{(\alpha_{1},\alpha_{2},\beta _{1},\beta_{2})} \bigl((t-x)^{2}(s-y)^{2};q_{n_{1}},q_{n_{2}},x,y \bigr)} \\& \qquad {} +\delta^{-1}_{n_{1}}\sqrt{ \mathcal{K}_{n_{1},n_{2},p_{1},p_{2}}^{(\alpha _{1},\alpha_{2},\beta_{1},\beta _{2})}\bigl((t-x)^{4}(s-y)^{2};q_{n_{1}},q_{n_{2}},x,y \bigr)} \\& \qquad {} +\delta^{-1}_{n_{2}}\sqrt{ \mathcal{K}_{n_{1},n_{2},p_{1},p_{2}}^{(\alpha _{1},\alpha_{2},\beta_{1},\beta _{2})}\bigl((t-x)^{2}(s-y)^{4};q_{n_{1}},q_{n_{2}},x,y \bigr)} \\& \qquad {}+\delta^{-1}_{n_{1}}\delta^{-1}_{n_{2}} \mathcal{K}_{n_{1},n_{2},p_{1},p_{2}}^{(\alpha_{1},\alpha_{2},\beta_{1},\beta_{2})} \bigl((t-x)^{2}(s-y)^{2};q_{n_{1}},q_{n_{2}},x,y \bigr) \Bigr)\omega_{\mathrm{mixed}}(D_{B} f;\delta _{n_{1}}, \delta_{n_{2}}). \end{aligned}$$

From Lemma 2, we observe that for $(t,s)\in(I_{1}\times I_{2})$, $(x,y)\in J^{2}$ and $i,j={1,2}$,

$$\begin{aligned}& K_{n_{1},n_{2},p_{1},p_{2}}^{(\alpha_{1},\alpha_{2},\beta_{1},\beta _{2})}\bigl((t-x)^{2i}(s-y)^{2j};q_{n_{1}},q_{n_{2}},x,y \bigr) \\& \quad = K_{n_{1},n_{2},p_{1},p_{2}}^{(\alpha _{1},\alpha_{2},\beta_{1},\beta_{2})}\bigl((t-x)^{2i};q_{n_{1}},x,y \bigr) K_{n_{1},n_{2},p_{1},p_{2}}^{(\alpha_{1},\alpha_{2},\beta_{1},\beta _{2})}\bigl((s-y)^{2j};q_{n_{2}},x,y \bigr). \\& \quad = K_{n_{1},p_{1}}^{(\alpha_{1},\beta_{1})}\bigl((t-x)^{2i};q_{n_{1}},x \bigr) K_{n_{2},p_{2}}^{(\alpha_{2},\beta_{2})}\bigl((s-y)^{2j};q_{n_{2}},y \bigr) \\& \quad \leq \frac{M_{1}}{[n_{1}]^{i}_{q_{n_{1}}}}\frac{M_{2}}{[n_{2}]^{j}_{q_{n_{2}}}} \end{aligned}$$

for some constants $M_{1},M_{2}>0 $.

Now, let $\delta_{n_{1}}=\frac{1}{[n_{1}]_{q_{n_{1}}}^{1/2}}$ and $\delta_{n_{2}}=\frac{1}{[n_{2}]_{q_{n_{2}}}^{1/2}}$,

4.5 $$\begin{aligned}& \bigl\vert T_{n_{1},n_{2},p_{1},p_{2}}^{(\alpha_{1},\alpha_{2},\beta_{1},\beta _{2})}(f;q_{n_{1}},q_{n_{2}},x,y)-f(x,y) \bigr\vert \\& \quad = 3\|D_{B}\|_{\infty}O \biggl( \frac {1}{[n_{1}]_{q_{n_{1}}}^{1/2}} \biggr) O \biggl(\frac{1}{[n_{2}]_{q_{n_{2}}}^{1/2}} \biggr) \\& \qquad {}+O \biggl(\frac{1}{[n_{1}]_{q_{n_{1}}}^{1/2}} \biggr)O \biggl(\frac {1}{[n_{2}]_{q_{n_{2}}}^{1/2}} \biggr) \omega_{\mathrm{mixed}}\bigl(D_{B} f;[n_{1}]_{q_{n_{1}}}^{-1/2},[n_{2}]_{q_{n_{2}}}^{-1/2} \bigr). \end{aligned}$$

Thus, we obtain the required result. □

Now, we illustrate the rate of convergence of the GBS operators (4.2) to certain functions by graphics. It is observed that when the values of $q_{1}$ and $q_{2}$ increase, the convergence of the GBS operator $T_{n_{1},n_{2},p_{1}p_{2}}^{ ( \alpha_{1},\alpha_{2},\beta _{1},\beta _{2} ) } ( f;q_{1},q_{2},x,y )$ to the function $f(x,y)$ becomes better.

### Example 4

**Figure 5:**
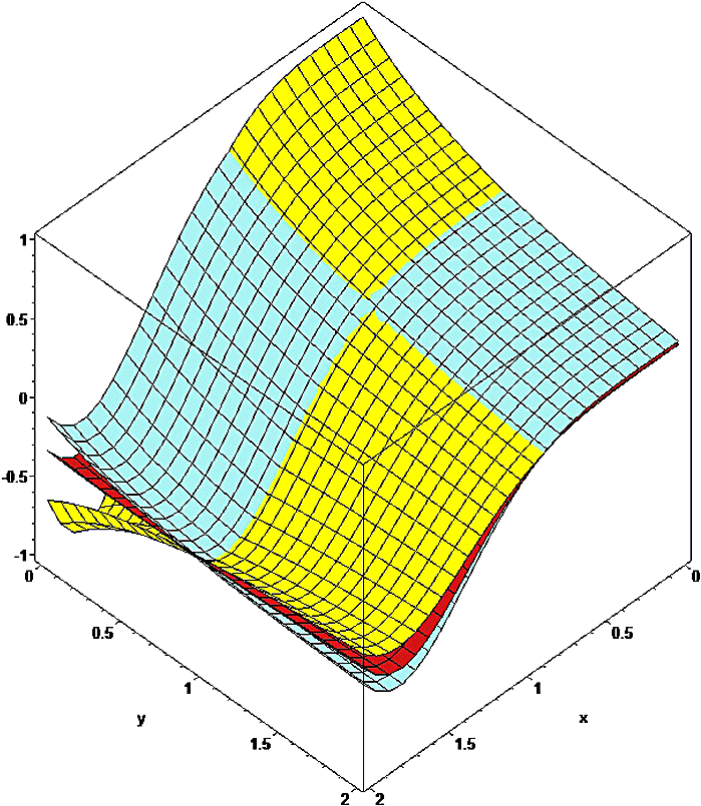
**Convergence of** $\pmb{T_{n_{1},n_{2},p_{1},p_{2}}^{(\alpha_{1},\alpha_{2},\beta_{1},\beta_{2})}(f;q_{1},q_{2},x,y)}$ **to** $\pmb{f(x,y)}$ **.**

Let $n_{1}=n_{2}=5$, $\alpha_{1}=5$, $\beta_{1}=6$, $\alpha _{2}=7$, $\beta_{2}=8$, $p_{1}=p_{2}=1$. For $q_{1}=0.45$, $q_{2}=0.50$ and $q_{1}=0.85$, $q_{2}=0.90$, the convergence of the GBS operators $T_{n_{1},n_{2},p_{1}p_{2}}^{ ( \alpha_{1},\alpha_{2},\beta _{1},\beta_{2} ) } ( f;q_{1},q_{2}, x,y )$ (turquoise, orange) to $f ( x,y ) =\cos ( x^{2} ) / (1+y ) $ (yellow) is shown in Figure 5.

Lastly, we compare the convergence of the operators $\mathcal{K}_{n_{1},n_{2},p_{1}p_{2}}^{ ( \alpha_{1},\alpha_{2},\beta _{1},\beta _{2} ) } ( f;q_{1},q_{2},x,y ) $ given by (3.1) and its GBS operators $T_{n_{1},n_{2},p_{1}p_{2}}^{ ( \alpha_{1},\alpha _{2},\beta _{1},\beta_{2} ) } ( f;q_{1},q_{2},x,y ) $ to some functions.

### Example 5

**Figure 6:**
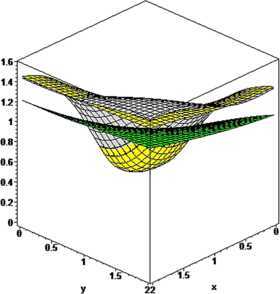
**The comparison of rate of convergence of** $\pmb{K_{n_{1},n_{2},p_{1},p_{2}}^{(\alpha_{1},\alpha_{2},\beta_{1},\beta_{2})}(f;q_{1},q_{2},x,y)}$ **and** $\pmb{T_{n_{1},n_{2},p_{1},p_{2}}^{(\alpha_{1},\alpha_{2},\beta_{1},\beta_{2})}(f;q_{1},q_{2},x,y)}$ **to** $\pmb{f(x,y)}$ **.**

**Figure 7:**
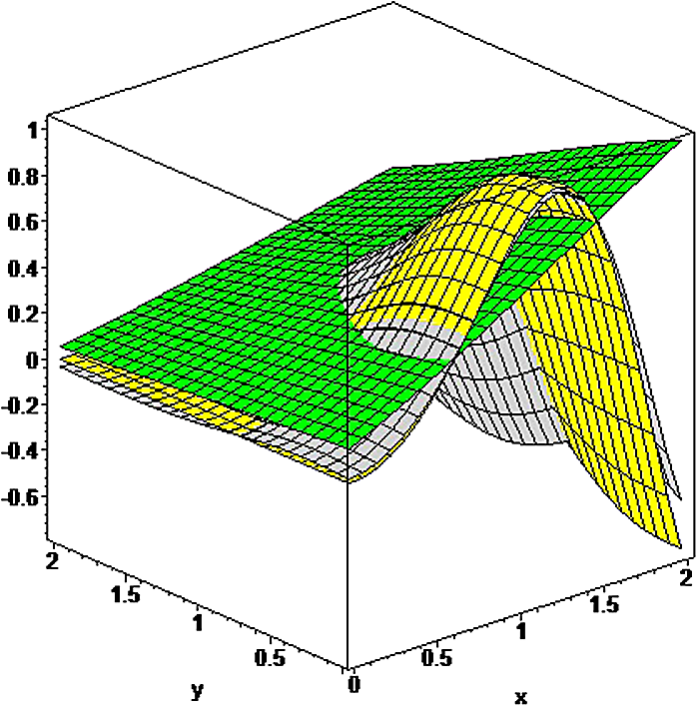
**The comparison of rate of convergence of** $\pmb{K_{n_{1},n_{2},p_{1},p_{2}}^{(\alpha_{1},\alpha_{2},\beta_{1},\beta_{2})}(f;q_{1},q_{2},x,y)}$ **and** $\pmb{T_{n_{1},n_{2},p_{1},p_{2}}^{(\alpha_{1},\alpha_{2},\beta_{1},\beta_{2})}(f;q_{1},q_{2},x,y)}$ **to** $\pmb{f(x,y)}$ **.**

**Figure 8:**
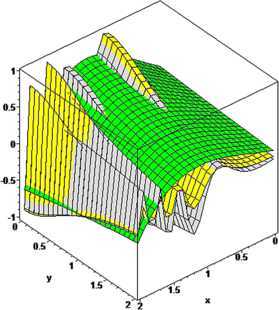
**The comparison of rate of convergence of** $\pmb{K_{n_{1},n_{2},p_{1},p_{2}}^{(\alpha_{1},\alpha_{2},\beta_{1},\beta_{2})}(f;q_{1},q_{2},x,y)}$ **and** $\pmb{T_{n_{1},n_{2},p_{1},p_{2}}^{(\alpha_{1},\alpha_{2},\beta_{1},\beta_{2})}(f;q_{1},q_{2},x,y)}$ **to** $\pmb{f(x,y)}$ **.**

For $n_{1},n_{2}=5$, $\alpha_{1}=2$, $\beta_{1}=3$, $\alpha _{2}=4$, $\beta_{2}=5$, $p_{1}=p_{2}=1$ and $q_{1}=0.75$, $q_{2}=0.80$, the comparison of convergence of the operators $\mathcal{K}_{n_{1},n_{2},p_{1}p_{2}}^{ ( \alpha_{1},\alpha_{2},\beta_{1},\beta_{2} ) } ( f;q_{1},q_{2},x,y ) $ (green) and $T_{n_{1},n_{2},p_{1}p_{2}}^{ ( \alpha_{1},\alpha_{2},\beta_{1},\beta_{2} ) } ( f;q_{1},q_{2},x,y ) $ (gray) to the functions $f ( x,y ) =\arctan ( x^{3}+y^{2} ) $, $f ( x,y ) =\sin ( x^{2} ) / ( 1+y^{3} ) $, $f ( x,y ) =\sin (3x^{3} ) / ( 1+y^{2} ) $ is illustrated, respectively, in Figures 6, 7, and 8. We observe that the rate of convergence of $T_{n_{1},n_{2},p_{1}p_{2}}^{ ( \alpha_{1},\alpha_{2},\beta _{1},\beta _{2} ) } ( f;q_{1},q_{2},x,y ) $ is better than the operator $\mathcal{K}_{n_{1},n_{2},p_{1}p_{2}}^{ ( \alpha_{1},\alpha _{2},\beta_{1},\beta_{2} ) } ( f;q_{1},q_{2},x,y ) $.
